# A Viscous DES‐AAV‐*Foxo1* Delivery System With High Transfection Efficiency for the Treatment of Corneal Endothelial Dysfunction by Restoring Mitochondria‐ER Contacts

**DOI:** 10.1002/advs.75464

**Published:** 2026-05-03

**Authors:** Hongran Zhao, Xiaoyu Li, Hongwei Wang, Zongyi Li, Qun Wang, Yangyang Zhang, Xu Jing, Xia Qi, Qingjun Zhou, Shengqian Dou, Lixin Xie

**Affiliations:** ^1^ Eye Institute of Shandong First Medical University Qingdao China; ^2^ State Key Laboratory Cultivation Base Shandong Key Laboratory of Eye Diseases Qingdao China; ^3^ School of Ophthalmology Shandong First Medical University Qingdao China; ^4^ Department of Microbiology Tumor and Cell Biology Karolinska Institute Stockholm Sweden

**Keywords:** corneal endothelial dysfunction, deep eutectic solvent (DES), FOXO1, gene delivery, mitochondria‐associated endoplasmic reticulum membranes (MAMs)

## Abstract

Corneal endothelial dysfunction is a major cause of global blindness, with an estimated 12.7 million patients awaiting corneal transplantation, and the severe shortage of donor grafts underscores the urgent need for non‐surgical therapies. Gene therapy offers a promising alternative, but is hindered by the limitations in existing delivery systems and the scarcity of validated molecular targets capable of reversing core pathophysiology. To address this, we first employed multi‐omics analysis and identified FOXO1 as a central and under‐explored therapeutic target for corneal endothelial dysfunction. In vivo FOXO1 overexpression effectively improved corneal endothelial function by preserving mitochondria‐associated endoplasmic reticulum membrane integrity and mitochondrial Ca^2^^+^ homeostasis, yet its therapeutic potential was limited by low transfection efficiency. To overcome this, we engineered an AAV‐*Foxo1* delivery system using a viscous choline chloride‐fructose‐based deep eutectic solvent (DES) as the carrier. The DES‐AAV‐*Foxo1* delivery system exhibited good biocompatibility, significantly prolonged anterior chamber retention, and enhanced transfection efficiency in corneal endothelial cells compared to conventional AAV delivery. Animal experiments confirmed that it effectively improved corneal endothelial pump activity and mitigates endothelial dysfunction in type 1 diabetes mellitus and Fuchs endothelial corneal dystrophy mouse models. Our findings demonstrated the therapeutic potential of DES‐AAV‐*Foxo1* delivery system for corneal endothelial disorders.

## Graphical abstract

High glucose triggers corneal endothelial dysfunction by impairing FOXO1‐mediated *ITPR1* transcription, leading to disrupted mitochondria‐associated membrane (MAM) integrity and defective ER‐to‐mitochondria Ca^2^^+^ transfer. This study develops an innovative viscous DES‐AAV‐*Foxo1* delivery system with enhanced transfection efficiency. This non‐invasive gene therapy approach successfully restores MAM integrity and corneal endothelial function, offering a highly promising therapeutic strategy for corneal blindness globally.

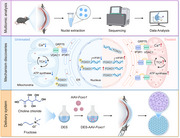

## 1 Introduction

Corneal endothelial dysfunction is a leading cause of global vision impairment and blindness [1], with an estimated 12.7 million people currently awaiting treatment [2]. Corneal endothelium can be damaged by various pathological conditions, such as Fuchs endothelial corneal dystrophy (FECD), inflammation, and invasive cataract and glaucoma surgery, leading to a decrease in corneal endothelial cell (CEnC) density [3, 4, 5, 6]. When the density decreases below the threshold of approximately 300–500 cells/mm^2^, endothelial decompensation occurs, resulting in corneal edema, bullous keratopathy, and ultimately irreversible vision loss [7], and corneal transplantation is thereby left as the remaining therapeutic option [8]. However, a huge disparity between the number of patients waiting for transplantation and the supply of corneal graft donors remain a critical challenge [6]. Although recent pharmacological strategies aimed at improving corneal endothelial functions have shown promise in preclinical studies, none have yet been widely implemented in routine clinical practice [9, 10, 11]. Therefore, there is an urgent need to elucidate the pathological mechanisms and key regulatory targets driving corneal endothelial dysfunction, and develop novel, effective, non‐surgical strategies to prevent or treat corneal endothelial disorders.

Gene therapy represents a promising therapeutic intervention for various ocular diseases [12, 13, 14], including those affecting the corneal endothelium [15, 16]. Intracameral injection is a direct and effective method for delivering AAV vectors to the CEnCs, yet its efficacy is substantially limited by rapid viral clearance due to aqueous humor circulation and significant reflux through the limbal puncture site [16]. Recent studies have reported various approaches to enhance the AAV transfection [17, 18], such as hydrogels encapsulation of AAV for promoting tissue wound healing [19], neuron injury [20], and myocardial infarction [21]. Nevertheless, the slow degradation kinetics of hydrogels poses a potential risk of occluding the iridocorneal angle in the anterior chamber. Alternative strategies like metal nanoparticles are also considered unsuitable for intracameral injection owing to risks of organ toxicity and suboptimal particle size [22, 23, 24]. Thus, it is imperative to develop a safe and effective AAV delivery system specifically engineered to overcome rapid clearance and achieve extended retention in the anterior chamber.

Deep eutectic solvents (DESs), first synthesized in 2003 by Abbott et al. [25], are low‐melting mixtures prepared by combining hydrogen bond donors and hydrogen bond acceptors through hydrogen bonding interactions [26]. Due to advantages such as low cost, availability, non‐toxicity, non‐flammability, biodegradability, tunability, and recyclability [27], DESs have been successfully applied in diverse fields including the extraction of natural products, biocatalysis, electrochemistry, nanomaterials, and organic reactions [28]. Notably, DESs can increase drug solubility, improve drug permeability, and enhance drug stability, showing great potential of DESs in drug delivery systems in recent years [29, 30]. Beyond delivering conventional drugs, DESs held significant potential as carriers for nuclei acids, such as plasmid DNA (pDNA), small interfering RNA (siRNA), and mRNA, which could improve the efficacy of gene therapies without altering their structures [31, 32, 33, 34]. To date, no studies have explored the use of DES as a carrier for AAV delivery, and represented a promising yet unreported direction for DESs in biomedicine.

**FIGURE 1:**
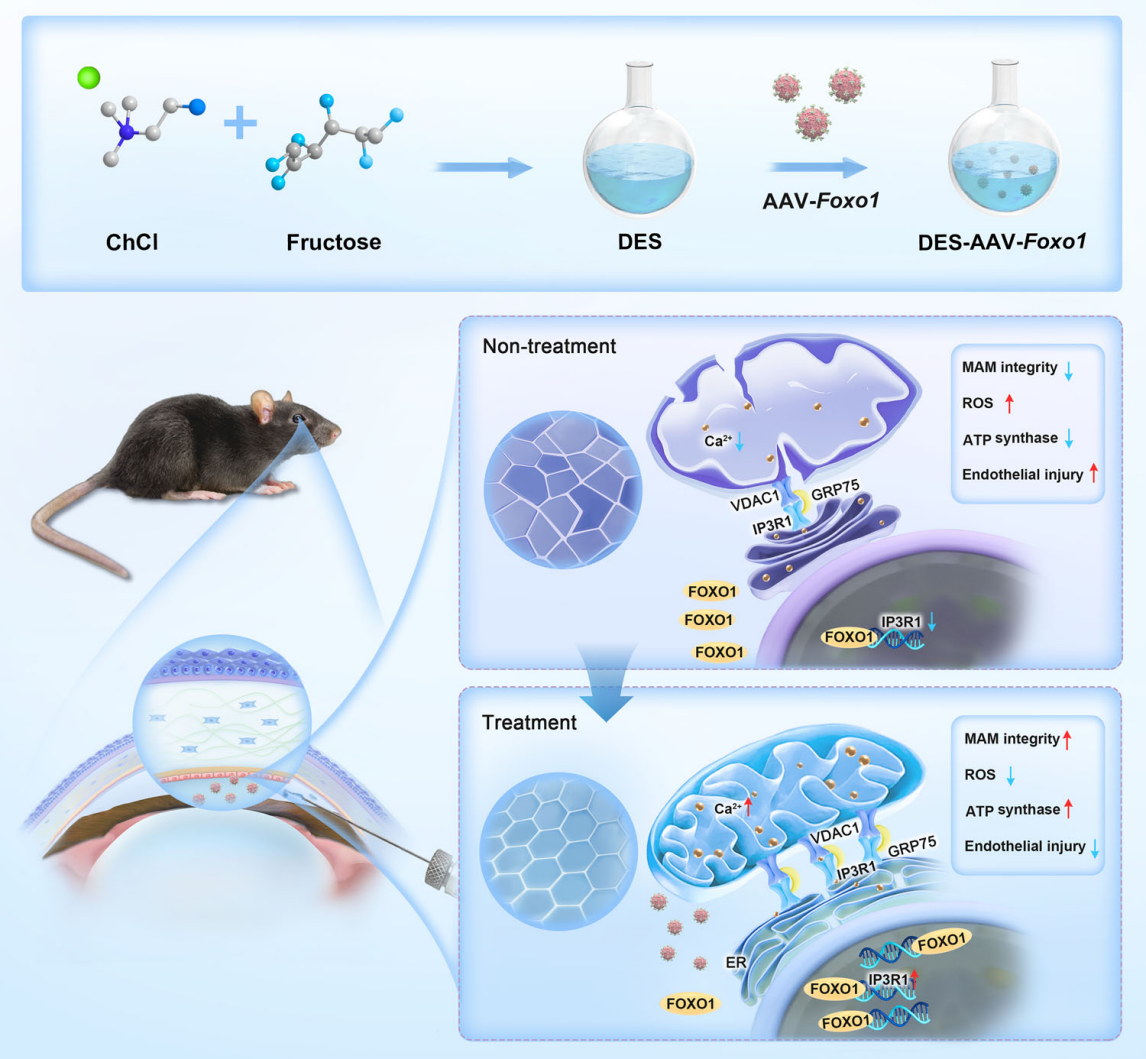
Schematic representation of the design and synthesis of a deep eutectic solvents‐based AAV delivery (DES‐AAV‐*Foxo1*) system for the treatment of corneal endothelial dysfunction by FOXO1 overexpression.

In this study, we identified FOXO1 as a potential therapeutic target of corneal endothelial dysfunction employing single‐nucleus RNA sequencing (snRNA‐seq) and single‐cell assay for transposase‐accessible chromatin sequencing (scATAC‐seq) in an established type 1 diabetes mellitus (T1DM) model. Functional validation confirmed that FOXO1 restoration through AAV delivery improves corneal endothelial functions by enhancing the integrity of mitochondria–endoplasmic reticulum (ER) contacts and maintaining mitochondrial Ca^2^^+^ homeostasis. To improve the transfection efficiency of AAV, we engineered a choline chloride‐fructose‐based DES, as an AAV delivery vehicle for the treatment of corneal endothelial dysfunction. Initially, AAV was encapsulated into the DES with stirring to prepared the delivery system. Then, we assessed its biosafety and transduction efficiency in mouse CEnCs in vivo. Finally, therapeutic effects of the DES‐AAV‐*Foxo1* system were evaluated in both T1DM and FECD mouse models (Figure 1).

## 2 Results and Discussion

### 2.1 ER and Mitochondrial Dysfunction in CEnCs of T1DM Mice

**FIGURE 2:**
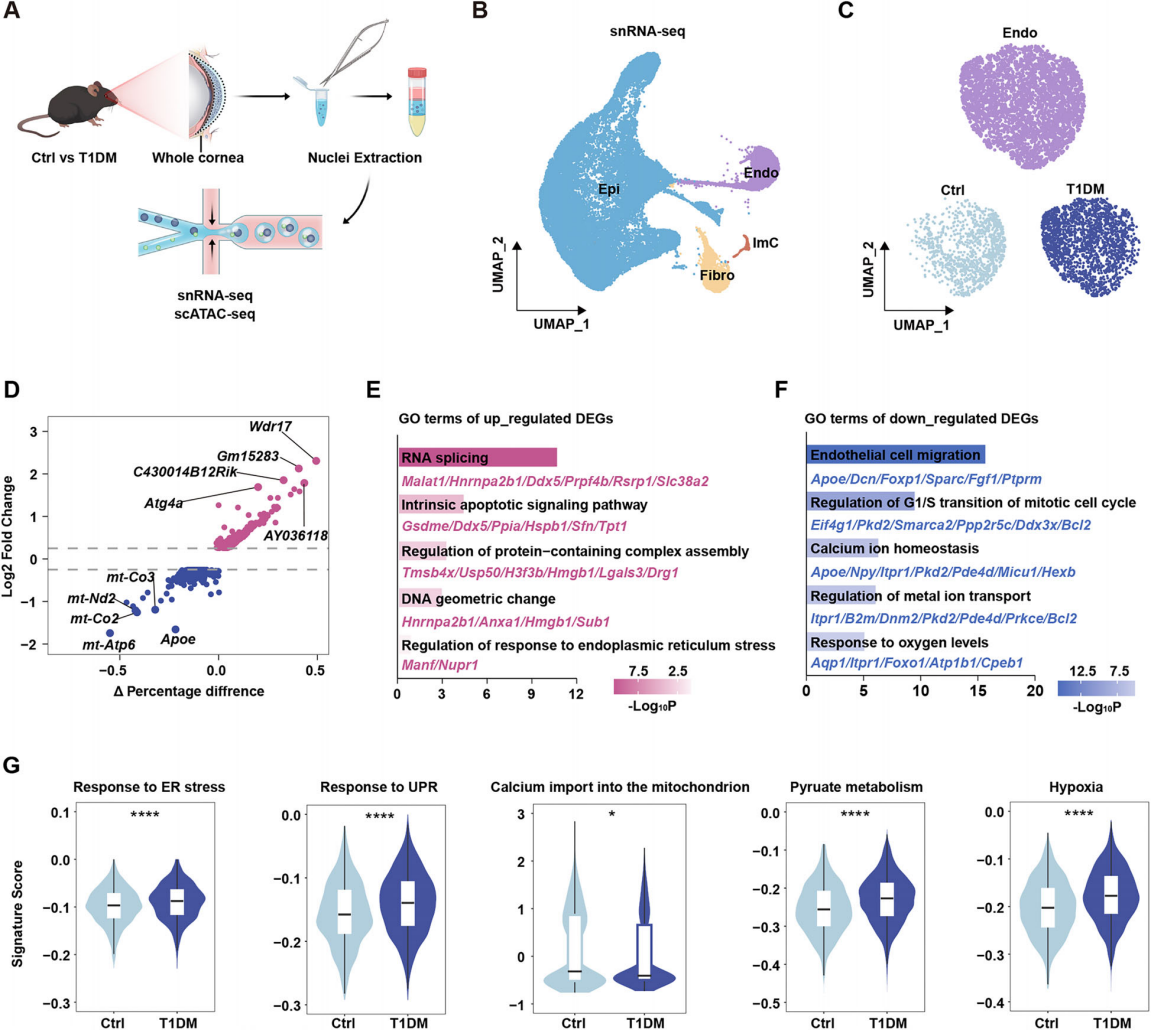
ER stress and impaired mitochondrial function in T1DM‐incuced corneal endothelial dysfunction. (A) Flow chart depicting the snRNA‐seq and scATAC‐seq workflow of this study. (B) Uniform Manifold Approximation and Projection (UMAP) plot colored by cell types in corneal snRNA‐seq data. Epi: Epithelial cells; Fibro: Stromal keratocytes; Endo: Endothelial cells; ImC: Immune cells. (C) UMAP plots depicting integrated (upper) and group‐stratified (lower) CEnCs. (D) Differentially expressed genes (DEGs) in CEnCs from T1DM mice compared with that in controls (ordered by p‐value). The names of the top 5 genes are labeled. (E,F) Representative gene ontology (GO) terms and genes enriched from DEGs of T1DM mouse CEnCs compared with controls. (G) Gene set scores of pathways related to the ER and mitochondria in T1DM mouse CEnCs and controls. UPR: Unfolded protein response. ^*^ *p* < 0.05; ^****^ *p* < 0.0001 (Wilcoxon rank‐sum test).

Given the critical importance of protecting the corneal endothelium [9], we employed an established mouse model of diabetes‐induced corneal endothelial dysfunction to investigate its mechanisms [35, 36]. First, we conducted snRNA‐seq on corneal tissues from T1DM and control mice (Figure 2A), and obtained a total of 33 369 nuclei after quality control (Figure S1A), annotated as epithelial cells (Epi), stromal keratocytes (Fibro), endothelial cells (Endo) and immune cells (ImC), according to representative marker genes (Figure 2B; Figure S1B,C and Dataset S1) [37, 38]. Among these, we subset 3066 CEnCs, accounting for approximately 9% of the total corneal cells (Figure 2C), for further exploration (Figure S1D). Further differentially expressed gene (DEG) analysis revealed the top 5 downregulated genes—including *mt‐Atp6*, *Apoe*, *mt‐Nd2*, *mt‐Co2*, and *mt‐Co3—*all of which are associated with mitochondrial process, suggesting impaired mitochondrial function (Figure 2D and Dataset S2). Gene ontology (GO) analysis demonstrated that the upregulated genes were associated with cell apoptosis, DNA geometric changes and ER stress (Figure 2E), whereas the downregulated genes were enriched in terms of response to oxygen levels and Ca^2+^ homeostasis (Figure 2F)—both of which are closely linked to mitochondrial function [39]. Further gene set score analysis also revealed an increase in ER stress‐related and hypoxia‐related signals (Figure 2G and Dataset S3). Specifically, elevated unfolded protein response (UPR) scores were indicative of disrupted ER proteostasis [40], while excessive pyruvate metabolism was found to perturb mitochondrial homeostasis through the accumulation of tricarboxylic acid (TCA) cycle intermediates and the concomitant production of reactive oxygen species (ROS) [41]. Additionally, hypoxia signals were coupled to mitochondrial dysfunction through a mutual reinforcement loop, further exacerbating cellular stress [42]. Collectively, our data revealed that ER stress and impaired mitochondrial function play a crucial role in diabetes‐induced corneal endothelial dysfunctions.

### 2.2 Impaired ER‐Mitochondria Interactions Underlie Diabetic Corneal Endothelial Dysfunction

**FIGURE 3:**
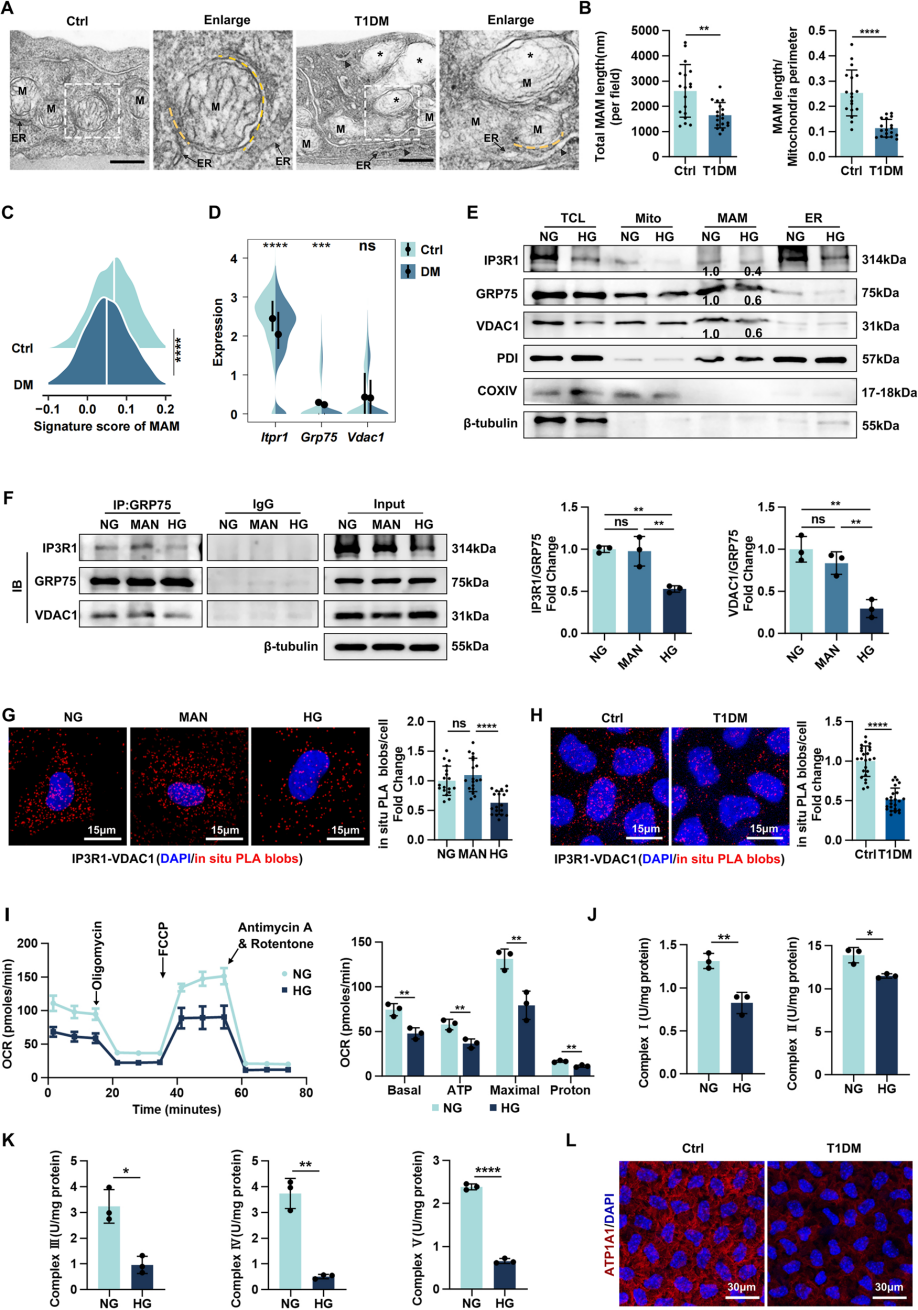
Disruption of ER‐mitochondria interactions and associated morphological changes underlie diabetes‐induced corneal endothelial dysfunction. (A) Representative transmission electron microscopy (TEM) images of CEnCs from control and T1DM mice (*n* = 3) showing alterations of the ultrastructures of the ER and mitochondria (labeled “M”), including ER degranulation, fragmentation and distortion (indicated as triangles), swollen mitochondria with cristae loss (asterisks), and preserved MAM interfaces (yellow dashed lines). Scale bar: 500 nm. (B) Quantification of the total length of the MAM per field and the ratio of MAM length to the mitochondrial perimeter (*n* = 6). (C) Gene set score analysis of MAM‐related genes between groups. (D) Expression levels of *Itpr1*, *Grp75* and *Vdac1* between groups in snRNA‐seq data. (E) WB analysis of IP3R1, GRP75 and VDAC1 in the MAM fractions of human CEnCs cultured under normal and high glucose (*n* = 3). TCL: total cell lysates; Mito: purified mitochondria; NG: normal glucose; HG: high glucose. (F) Co‐IP assays were used to evaluate the interaction between GRP75 and IP3R1/VDAC1 in human CEnCs cultured under different conditions (*n* = 3). MAN: Mannitol. (G) PLA assay showing the interaction between IP3R1 and VDAC1 in human CEnCs cultured under different conditions (*n* = 3). Scale bar: 15 µm. (H) PLA assay showing the interaction between IP3R1 and VDAC1 in the CEnCs of control and T1DM mice (*n* = 3). Scale bar: 15 µm. I) Mitochondrial oxygen consumption rate (OCR) measurements were performed to assess basal respiration, ATP‐dependent respiration, maximal respiration, and proton leakage in NG‐ and HG‐treated CEnCs (*n* = 3). (J,K) The activity of mitochondrial respiratory chain (MRC) complexes I‐V in isolated mitochondria from NG and HG treated human CEnCs (*n* = 3). (L) Corneal whole‐mount staining showing ATP1A1 expression in control and T1DM mouse CEnCs (*n* = 3). Scale bar: 30 µm. Student's *t*‐test (B, E, H, I, J, K), wilcoxon rank‐sum test (C,D) and one‐way ANOVA (F,G) were used. ^*^ *p* < 0.05; ^**^ *p* < 0.01; ^***^ *p* < 0.001; ^****^ *p* < 0.0001; ns, not significant.

Prompted by these findings, we used transmission electron microscopy (TEM) to intuitively observe the ultrastructures of the ER and mitochondria. Morphologically, the ER lumen in the CEnCs of mice with T1DM appeared swollen and convoluted, with the loss of surface granules. Moreover, the mitochondria exhibited dense packing, swelling and loss of cristae (Figure 3A). Strikingly, mitochondrial‐ER contacts were significantly reduced in the CEnCs of T1DM mice (Figure 3A). Quantitative assessment further revealed that both the total mitochondria‐associated endoplasmic reticulum membrane (MAM) length per field and the ratio of MAM‐to‐mitochondrial perimeter ratio were markedly decreased compared to those of control mice (Figure 3B), which were corroborated at the molecular level by significantly lower signature scores of MAM‐related genes in the DM group (Figure 3C and Dataset S3). The MAM, ER‐mitochondria tethering structure, enables Ca^2^^+^ transfer via the IP3R1‐GRP75‐VDAC1 axis to regulate cellular energy homeostasis [43, 44]. Accordingly, both *Itpr1* and *Grp75* levels were significantly downregulated in DM mice (Figure 3D).

To further investigate the underlying mechanisms, we quantified the expression of inositol 1,4,5‐trisphosphate receptor type 1 (IP3R1), glucose‐regulated protein 75 (GRP75), and Voltage‐dependent anion channel 1 (VDAC1) at the MAM interface using subcellular fractionation of cultured human CEnCs. Results showed significantly lower levels of all three proteins in the MAM of high glucose (HG)‐treated CEnCs compared to normal glucose (NG) controls (Figure 3E and Figure S2A). Co‐IP assays further revealed a marked reduction in GRP75 binding to both IP3R1 and VDAC1 under HG conditions, compared to NG and mannitol (MAN) control groups (Figure 3F). Consistent with these findings, protein expression of IP3R1, GRP75 and VDAC1 was also decreased in CEnCs from T1DM mice (Figure S2B). Moreover, in situ proximity ligation assay (PLA) demonstrated a significant loss of IP3R1‐VDAC1 interaction in both HG‐treated cells and diabetic mouse CEnCs (Figure 3G,H). We also employed fluorescent trackers targeting organelles as an additional strategy to investigate changes in ER‐mitochondria interactions, and observed significantly reduced colocalization in HG‐treated CEnCs compared to NG and MAN controls, as indicated by a lower Pearson's coefficient (Figure S2C,D).

To systematically assess the functional impact of MAM disruption in diabetic CEnCs, we measured mitochondrial Ca^2^^+^ uptake and metabolic function. HG treatment significantly reduced mitochondrial Ca^2^^+^ levels (Figure S2E), indicating impaired Ca^2+^ transfer from ER to mitochondria. Given the dependence of tricarboxylic acid (TCA) cycle dehydrogenase activity on mitochondrial Ca^2^^+^ availability, this reduction was accompanied by a pronounced suppression of mitochondrial respiration [45]. Consistently, oxygen consumption rate (OCR) results revealed a marked decrease in basal, ATP‐linked, maximal respiration and proton leak (Figure 3I; Figure S2F). These alterations indicated compromised basal energy supply, reduced mitochondrial ATP production capacity, and impaired ability to adapt to increased energetic demand. Furthermore, the concurrent decrease in proton leak reflects a generalized suppression of electron transport chain (ETC) flux [46]. Importantly, the activities of mitochondrial respiratory chain (MRC) complexes I‐V were significantly decreased in CEnCs exposed to HG compared with NG controls (Figure 3J,K), providing functional evidence that impaired mitochondrial Ca^2+^ handling compromises ETC efficiency and oxidative metabolism. Consistent with the energy deficit, T1DM mouse CEnCs exhibited reduced Na^+^/K^+^‐ATPase expression and increased central corneal thickness (CCT) (Figure 3L; Figure S2G–I). Collectively, these profound bioenergetic deficits leave CEnCs unable to meet the high ATP demands required for their critical pump functions, ultimately driving corneal endothelial dysfunction.

### 2.3 Role of FOXO1 in Promoting MAM Formation and Alleviating Corneal Endothelial Dysfunction

**FIGURE 4:**
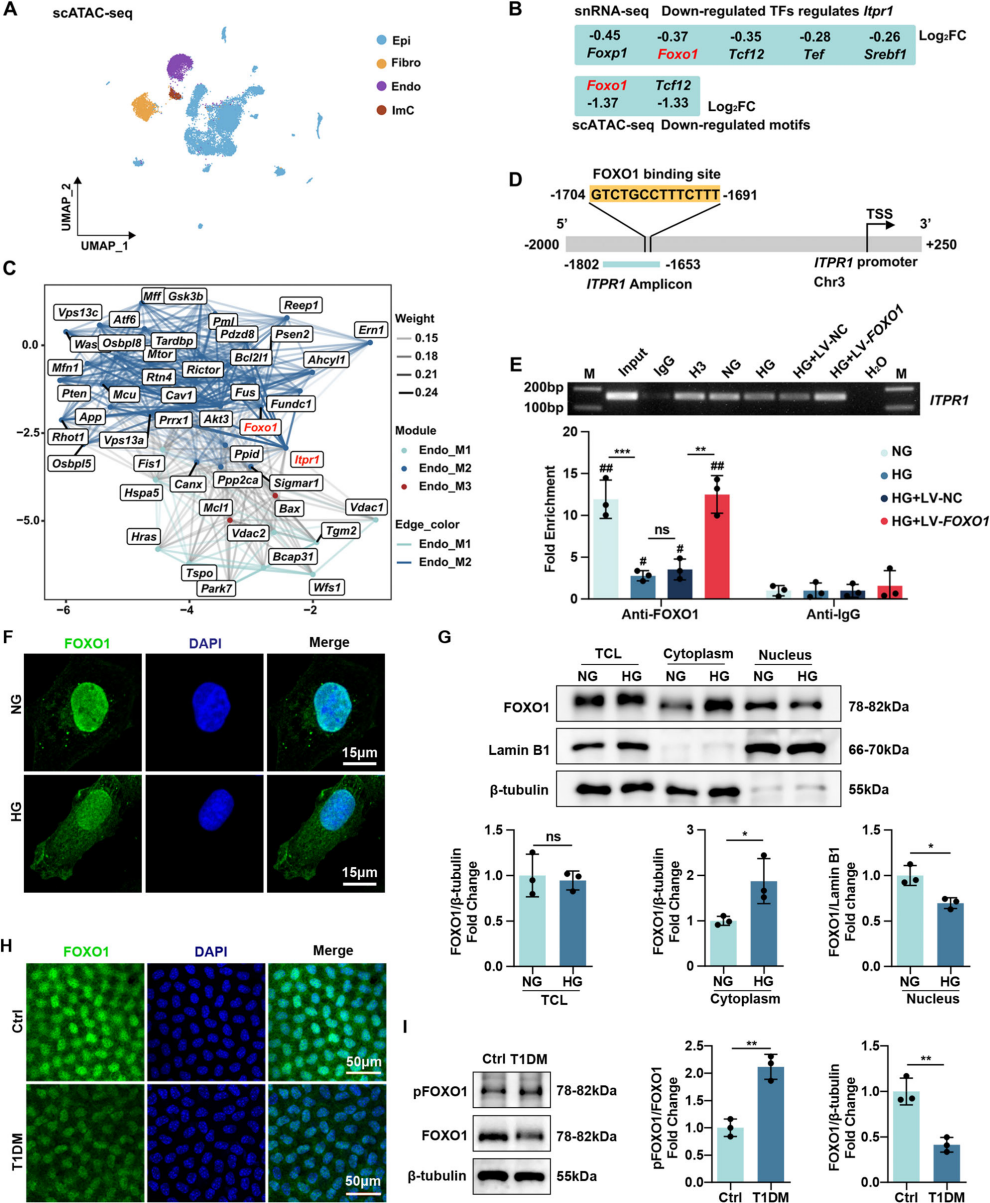
FOXO1 is the key transcriptional regulator of MAM formation. (A) UMAP plot colored by cell types in scATAC‐seq data. Epi: Epithelial cells; Fibro: stromal keratocytes; Endo: Endothelial cells; ImC: Immune cells. (B) The snRNA‐seq and scATAC‐seq datasets predicted that *Foxo1* is a potential upstream transcription factor of *Itpr1*. (C) hdWGCNA analysis showing the co‐expression network between *Foxo1* and MAM‐related genes. (D) Schematic diagram showing the potential FOXO1 binding site in the *ITPR1* promoter region. (E) ChIP assays verified the binding of FOXO1 to the *ITPR1* promoter region in human CEnCs (*n* = 3). LV: Lentivirus. (F) Subcellular localization of FOXO1 in human CEnCs treated with NG or HG for 12 h (*n* = 3). Scale bar: 15 µm. (G) WB analysis of FOXO1 protein levels in whole cell lysates and in the cytoplasm and nucleus of human CEnCs treated with NG or HG (*n* = 3). TCL: total cell lysates. (H) Corneal whole‐mount staining showing FOXO1 expression in the CEnCs of control mice and mice with T1DM (*n* = 3). Scale bar: 50 µm. (I) WB analysis of pFOXO1 and FOXO1 protein levels in mouse CEnCs (*n* = 3). One‐way ANOVA (E) and Student's *t*‐test (G,I) were used. ^*^ *p* <0.05; ^**^ *p* < 0.01; ^***^ *p* < 0.001; ns, not significant. ^#^, compared with the corresponding IgG (Student's *t*‐test). ^#^ *p* < 0.05; ^##^ *p* < 0.01.

To elucidate the underlying mechanisms and core transcription factors (TFs) governing MAM formation, we performed the SCENIC analysis and found that five TF regulons—*Foxp1*, *Foxo1*, *Srebf1*, *Tcf12* and *Tef*—were significant down‐regulated in diabetic CEnCs and predicted to regulate the ER calcium channel IP3R1 [47, 48, 49]. Subsequent differential accessibility analysis of scATAC‐seq data (Figure 4A; Figure S3A,B and Dataset S4) revealed the down‐regulated peaks (Dataset S5) and identified two potential binding domains, *Foxo1* and *Tcf12*. Of these, *Foxo1*, an essential member of the forkhead box protein O family [50, 51], was notably identified by both approaches and exhibited a substantial fold change (Figure 4B; Figure S3C,D). Furthermore, hdWGCNA placed *Foxo1* within the co‐expression modules enriched for MAM‐related genes, including *Itpr1* (Figure 4C). Consistently, ChIP assays confirmed FOXO1 occupancy at a region located 1653 to 1802 bp upstream of the *ITPR1* transcription start site (TSS) (Figure 4D,E; Figure S4A). In addition, compared with the control shRNA scramble group, CEnCs transfected with lentivirus‐mediated shRNA *FOXO1* showed a decreased expression of IP3R1 and enrichment at the promoter region of *ITPR1* (Figures S4A and S5A,B). Furthermore, knockdown *ITPR1* did not affect either total or nuclear FOXO1 protein levels in human CEnCs (Figures S4A and S6A). Importantly, rescue experiments revealed that *ITPR1* knockdown abolished the protective effects of FOXO1 overexpression, as FOXO1 overexpression failed to restore MAM formation and mitochondrial Ca^2^^+^ loading in *ITPR1* knockdown cells (Figure S6B–F). Together, these findings demonstrate that FOXO1 exerts its regulatory role on MAM formation and mitochondrial Ca^2^^+^ homeostasis in an IP3R1‐dependent manner.

Since phosphorylation is known to mediate FOXO1 nuclear shuttling, we examined the phosphorylation of FOXO1 at Ser256, which directly influences its translocation [52]. As expected, HG treatment significantly increased the level of phosphorylated FOXO1 at multiple time points in CEnCs (Figure S7A–C). We then conducted immunofluorescence staining in CEnCs to visualize the subcellular distribution of FOXO1 under HG stress. As shown in Figure 4F,G, FOXO1 was predominantly found in the nucleus under NG conditions, while HG treatment decreased nuclear FOXO1 expression and increased cytosolic FOXO1 expression, implying that HG promoted FOXO1 nuclear exclusion. Notably, phosphorylated FOXO1 expression was significantly increased and nuclear FOXO1 expression was significantly decreased in the CEnCs of T1DM mice (Figure 4H,I). These results suggest that HG treatment promotes the phosphorylation of FOXO1 at Ser256, facilitating its translocation to the cytoplasm and simultaneously affecting its transcriptional function.

**FIGURE 5:**
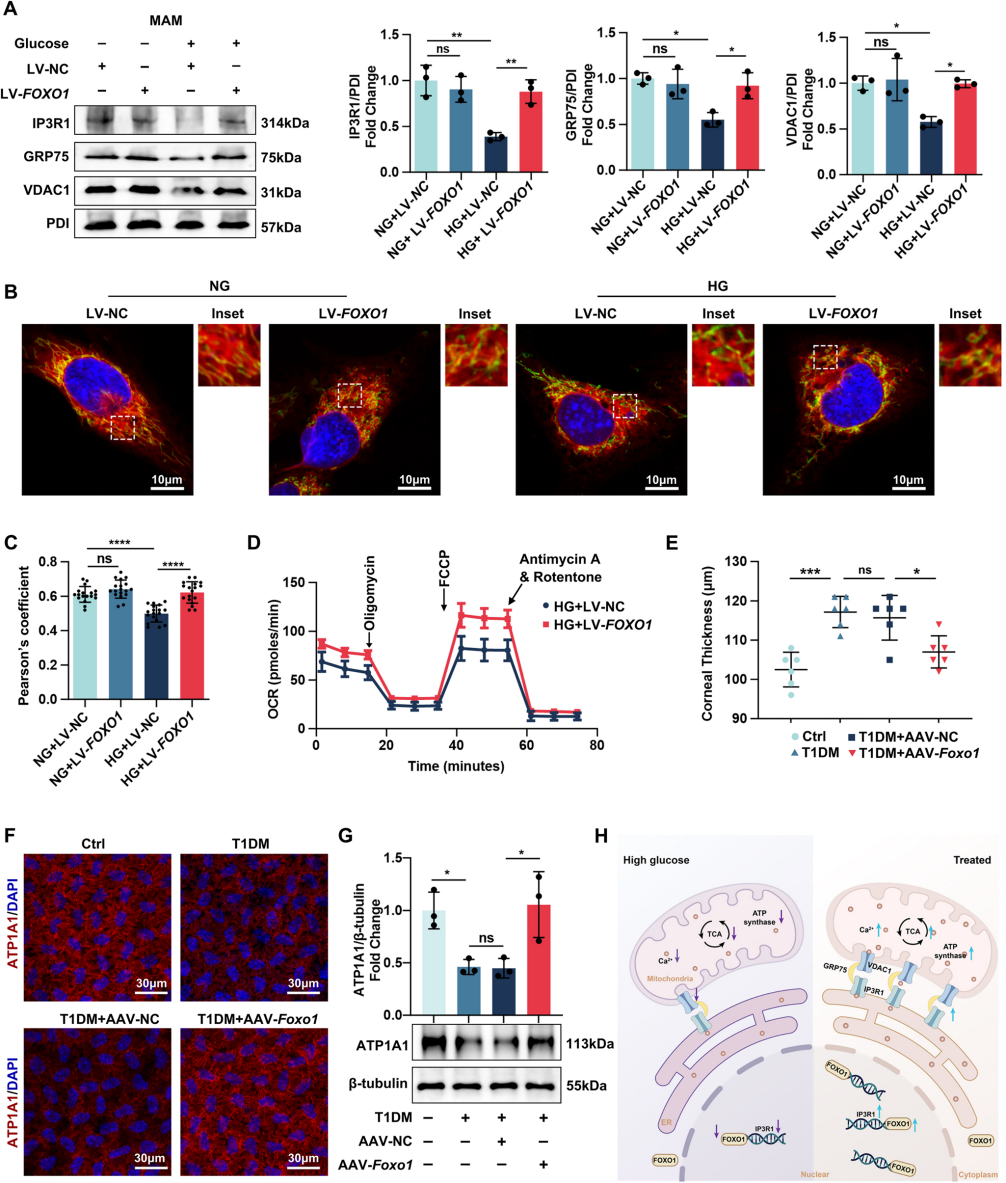
FOXO1 restoration alleviates diabetes‐induced corneal endothelial dysfunction. (A) WB analysis of IP3R1, GRP75 and VDAC1 in MAM fractions of human CEnCs from indicated groups (*n* = 3). LV: Lentivirus. (B) Confocal images displaying the associations between ER (ER‐Tracker Red) and mitochondria (Mito‐Tracker Green) in cultured human CEnCs. Scale bar: 10 µm. (C) Correlation analysis of ER and mitochondrial contacts using Pearson's coefficient (*n* = 3). (D) OCR measurements were performed to assess the mitochondrial function of CEnCs after *FOXO1* overexpression (*n* = 3). (E) Quantification of central corneal thickness (CCT) in different groups (*n* = 6). (F) Corneal whole‐mount staining showing the ATP1A1 expression in mouse CEnCs (*n* = 3). Scale bar: 30 µm. (G) WB analysis of ATP1A1 protein levels in mouse CEnCs (*n* = 3). (H) Schematic illustration showing FOXO1 mitigates diabetes‐induced corneal endothelial dysfunction by enhancing Ca^2+^ transfer at the ER‐mitochondria interface. AAV: Adeno‐associated virus. One‐way ANOVA (A,C,E,G) was used. ^*^ *p* < 0.05; ^**^ *p* < 0.01; ^***^ *p* < 0.001; ^****^ *p* < 0.0001; ns, not significant.

To evaluate the effects of FOXO1 on MAM formation in CEnCs, we overexpressed FOXO1 in HG‐treated CEnCs (Figure S8A), and found that protein levels of IP3R1, GRP75, and VDAC1 in MAM fractions were significantly elevated, suggesting that FOXO1 counteracted MAM disruption in HG‐induced corneal endothelial dysfunction (Figure 5A). Consistent with this, co‐staining with Mito‐Tracker and ER‐Tracker revealed that *FOXO1*‐overexpressing significant enhanced mitochondria‐ER contacts in CEnCs cultured in the HG condition (Figure 5B,C). In vivo, diabetic mice transduced with AAV‐*Foxo1* (Figure S8E) presented alleviation of CEnC histopathological changes. TEM further confirmed a notable increase in the MAM area in the CEnCs of AAV‐*Foxo1* treatment mice relative to controls (Figure S8B,C). Moreover, *FOXO1* overexpression in HG‐cultured CEnCs significantly increased mitochondrial Ca^2+^ levels (Figure S8D) and increased the oxygen consumption rate (OCR) (Figure 5D; Figure S8F). Notably, in diabetic mice, AAV‐*Foxo1* delivery resulted in markedly reduced CCT (Figure 5E) and increased Na^+^/K^+^‐ATPase expression (Figure 5F,G), suggesting a restoration of pump function. Collectively, these findings demonstrate that AAV‐*Foxo1* therapy effectively restores MAM integrity and improves mitochondrial function in diabetic corneal endothelial dysfunction (Figure 5H).

To investigate the broader transcriptional effect of FOXO1 overexpression, we conducted qPCR to assess the expression of key genes involved in mitochondrial function and homeostasis. The results revealed that Ca^2+^ transport‐associated genes in MAM, including *ITPR1*, *GRP75*, and *VDAC1*, were significantly upregulated following *FOXO1* overexpression (Figure S9A,B). In addition, genes encoding components mitochondrial respiratory chain (*MT‐ND2*, *NDUFA9*, *MT‐CYB*, *MT‐CO2*, *MT‐ATP6*, and *MT‐ATP8*) [53], as well as key regulators of mitochondrial biogenesis and dynamics (*NRF1*, *NFE2L2*, *TFAM*, *YME1L1*, *FIS1*, *DNM1L*, and *PINK1*) were significantly upregulated [54, 55, 56], whereas genes associated with mitochondrial outer membrane permeability and apoptosis (*BAX*, *BAK1*) were markedly downregulated (Figure S9C) in *FOXO1*‐overexpressing CEnCs [57]. Collectively, these results suggest the critical role of FOXO1 in repairing HG‐induced mitochondrial damage at the transcriptional level. We then performed bulk RNA‐seq of CEnCs transfected with *FOXO1*‐overexpressing lentivirus under HG conditions. A total 327 DEGs were identified in the *FOXO1*‐overexpression group and subsequently subjected to GO and KEGG enrichment analyses (Figure S10A,B and Dataset S6). The results revealed that the upregulated genes were not only enriched in the FOXO signaling pathway highlighted in this study, but were also associated with pathways related to cell junction. In contrast, the downregulated genes were mainly enriched in lipid metabolic processes (Figure S10C,D). These findings suggest that FOXO1 overexpression may also participate in maintaining intercellular junction integrity, as well as modulating lipid metabolism, which will be further investigated in future studies.

### 2.4 FOXO1‐Mediated Pump Function Enhancement Across Models of Corneal Endothelial Dysfunction

**FIGURE 6:**
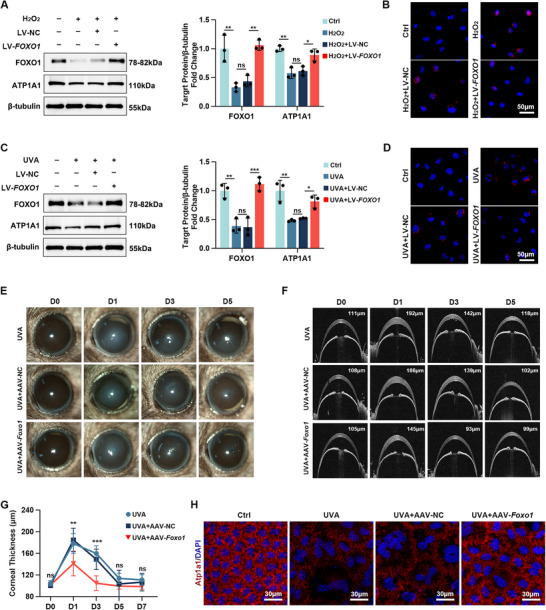
FOXO1 overexpression improves pump function across various corneal endothelial dysfunction models. (A) Western blot analysis of FOXO1 and ATP1A1 in H_2_O_2_‐induced oxidative stress models across indicated groups (*n* = 3). LV: Lentivirus. (B) MitoSOX staining (red) of mitochondrial ROS in H_2_O_2_‐treated CEnCs (*n* = 3). Scale bar, 50 µm. (C) Western blot analysis of FOXO1 and ATP1A1 protein levels UVA‐induced FECD models across indicated groups (*n* = 3). (D) MitoSOX staining (red) of mitochondrial ROS in UVA‐treated CEnCs (*n* = 3). Scale bar, 50 µm. (E) Representative slit‐lamp images showing the changes in pre‐ or post‐UVA treated corneas in different groups (*n* = 6). AAV: Adeno‐associated virus. (F,G) Representative OCT images of the mouse cornea (F) and quantification of CCT (G) (*n* = 6). ^*^ represents the difference between UVA+AAV‐NC and UVA+AAV‐*Foxo1* groups. (H) Corneal whole‐mount staining showing the ATP1A1 expression in mouse CEnCs (*n* = 4). Scale bar: 30 µm. One‐way ANOVA (A, C, G) were used. ^*^ *p* < 0.05; ^**^ *p* < 0.01; ^***^ *p* < 0.001; ns, not significant.

Given the protective role of FOXO1 in diabetes‐induced corneal endothelial dysfunction, we sought to determine whether its protective effects are generalizable across distinct disease models that share oxidative stress‐driven corneal endothelial injury [3, 58, 59, 60, 61, 62, 63]. To this end, we employed multiple complementary experimental models representing both chronic and acute oxidative stress conditions, and further focused on FECD—one of the leading indications for corneal transplantation—which is characterized by progressive loss of CEnCs and subsequent corneal edema leading to vision loss [3, 64]. Its pathogenesis is known to be driven by genetic and environmental factors, with ultraviolet A (UVA) exposure recognized as critical environmental triggers [3, 65]. Thus, we used a previously reported FECD mouse model induced by UVA irradiation to recapitulates the morphological and molecular changes of FECD [66]. Consistent with this notion, our results revealed that FOXO1 downregulation is a common molecular event in corneal endothelial damage, observed in both UVA‐induced FECD model (Figure 6A) and H_2_O_2_‐induced oxidative stress model in vitro (Figure 6C). *FOXO1* overexpression consistently mitigated oxidative stress and enhanced pump function by reducing reactive oxygen species (ROS) levels (Figure 6B,D) and increased Na^+^/K^+^‐ATPase expression (Figure 6A,C) across the different endothelial dysfunction models. In a mouse model of FECD induced by 500 J/cm^2^ UVA radiation, AAV‐*Foxo1* treatment conferred significant therapeutic benefits, alleviating corneal edema at day 1 and day 3 post‐exposure (Figure 6E–G) and enhanced pump function (Figure 6H). Taken together, these findings underscore FOXO1 as a crucial protective regulator of corneal endothelial homeostasis under diverse pathological insults.

### 2.5 Identification of DES as a Promising Carrier for AAV Delivery in Anterior Chamber

**FIGURE 7:**
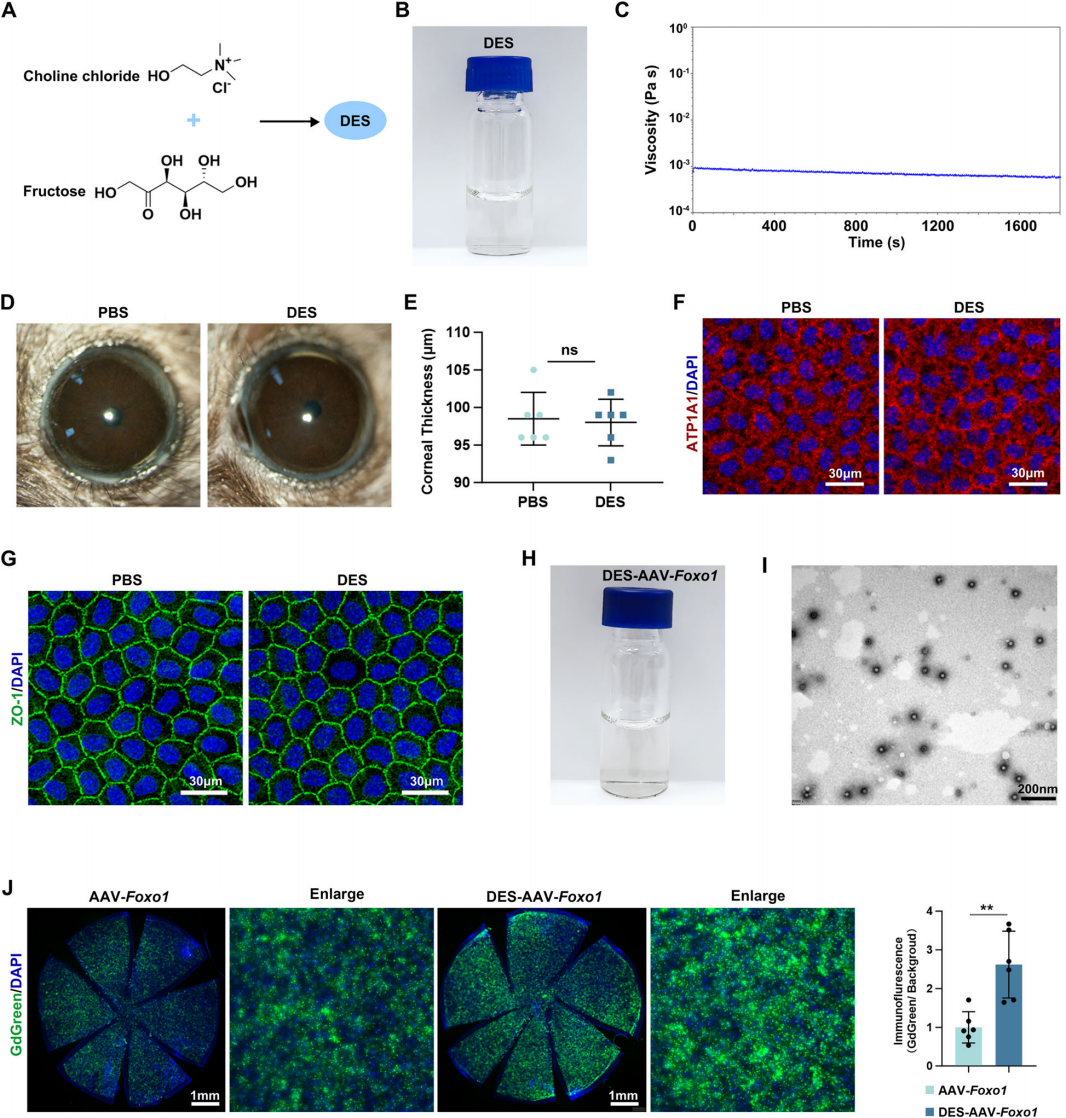
Characterization and safety of DES and the transfection efficiency of DES‐AAV‐*Foxo1* system. (A) Illustration of DES synthesis using choline chloride and fructose. (B) Visual examination of the DES (24°C). (C) Viscosity measurement of DES. (D) Representative slit‐lamp images of corneas from PBS‐ and DES‐treated mice. (E) Quantification of CCT in PBS‐ and DES‐AAV‐*Foxo1*‐treated mice (*n* = 6). (F,G) Corneal whole‐mount staining showing the expression of ATP1A1 and ZO‐1 in mouse CEnCs in different groups (*n* = 4). Scale bar: 30 µm. (H) Visual examination of the DES‐AAV‐*Foxo1* (24°C). AAV: Adeno‐associated virus. (I) TEM images showing the ultrastructure of AAV‐*Foxo1* in DES. Scale bar: 200 nm. (J) Immunofluorescence images of mouse corneal whole mounts and quantification of GdGreen fluorescence intensity in the CEnCs at 2 weeks after AAV‐*Foxo1* or DES‐AAV‐*Foxo1* infection (*n* = 6). Scale bar: 1 mm. Student's *t*‐test (E,G) were used. ^**^ *p* < 0.01; ns, not significant.

Efficient transduction of AAV into the anterior chamber remains a significant technical challenge. Traditional intracameral injection, even performed with microsyringe injection, substantial viral leakage from the corneal margin and aqueous humor outflow drastically diminishes the actual delivery efficiency [16, 67]. To compensate, studies usually use a high viral load of up to 1–5 × 10^9^ vg [68, 69], which raises concerns about potential immunogenic risks. To address these issues, we explored deep eutectic solvents (DES), a class of materials known for their favorable biosafety profile—including low toxicity and high biocompatibility—as well as tunable viscosity [28, 70], which aids in reducing solution leakage. In this study, a transparent, viscous DES was synthesized using choline chloride and fructose as precursors (Figure 7A–C). Safety evaluation via intracameral injection revealed no difference in corneal transparency, CCT, or the expression of Na^+^/K^+^‐ATPase and ZO‐1 in CEnCs between the PBS‐ or DES‐injected groups (Figure 7D–G), indicating that DES did not affect corneal thickness, pump function or barrier integrity. Furthermore, TEM result showed that the normal morphology of AAV, indicating that DES preserved the ultrastructural integrity of the virus without compromising the capsid structure (Figure 7H,I).

When the AAV dosage was reduced to 2.5 × 10^8^ vg, the DES‐encapsulated AAV‐*Foxo1* still achieved a significantly higher transduction efficiency within the corneal endothelial cells (Figure 7J; Figure S11A), and markedly improved corneal transparency, upregulated Na^+^/K^+^‐ATPase expression and preserved endothelial cell density compared to the unencapsulated AAV‐*Foxo1* group (Figure S11B–E). Together, our findings establish DES‐based release system as a robust strategy to overcome the key limitations of traditional intracameral AAV delivery by ensuring viral retention and enabling high‐efficiency transduction with a lower viral load.

### 2.6 In Vivo Treatment of Corneal Endothelial Dysfunction Using DES‐AAV‐*Foxo1* Delivery System

**FIGURE 8:**
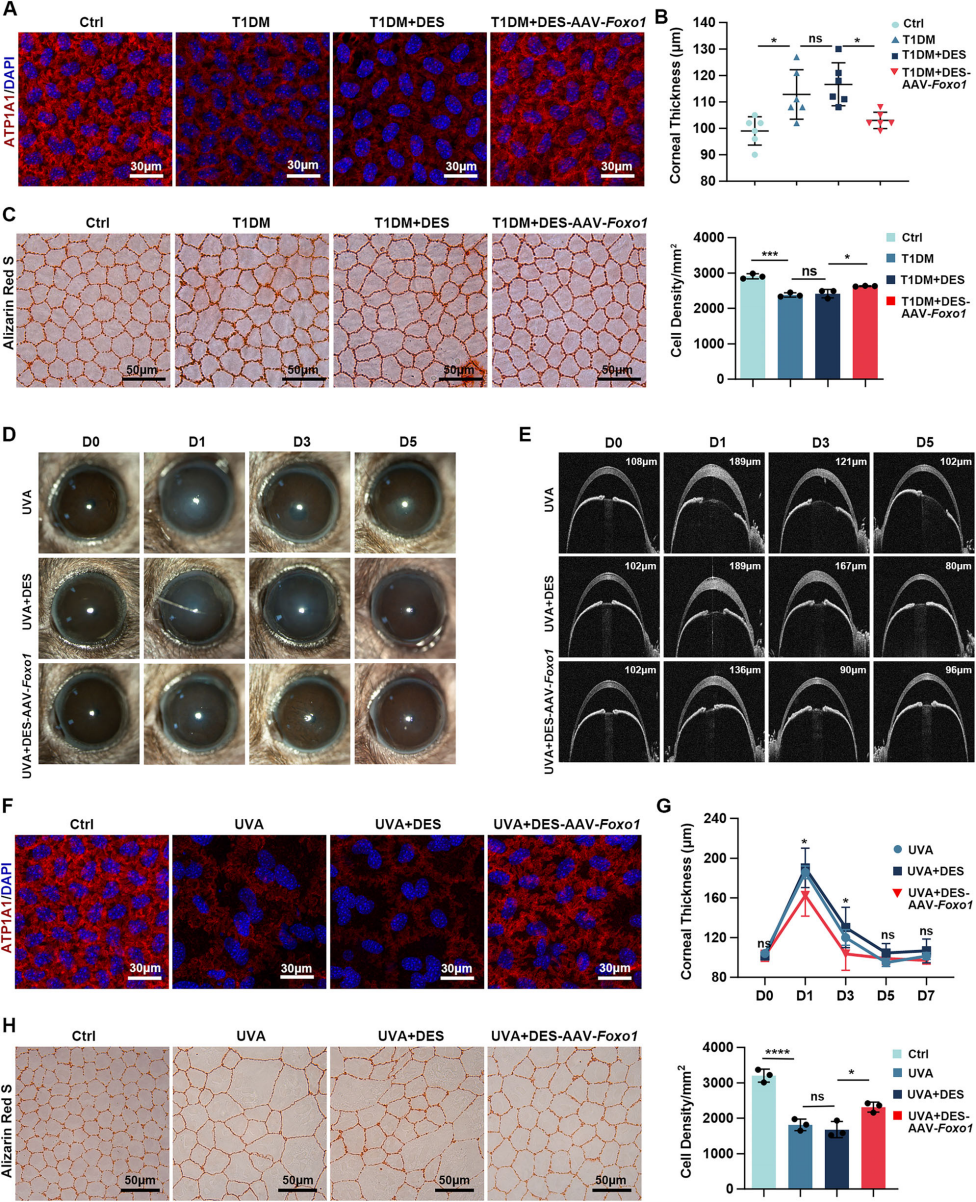
DES‐AAV‐*Foxo1* improves endothelial pump function and cell density in T1DM and FECD mouse models. (A) Corneal whole‐mount staining showing the ATP1A1 expression in mouse CEnCs of Ctrl, T1DM, T1DM treated with DES and T1DM treated with DES‐AAV‐*Foxo1* groups (*n* = 4). Scale bar: 30 µm. AAV: Adeno‐associated virus. (B) Quantification of CCT in different groups (*n* = 6). (C) Images of Alizarin Red‐stained of mouse CEnCs and quantification of cell density (*n* = 3). Scale bar: 50 µm. (D) Representative slit‐lamp images showing the changes in pre‐ or post‐UVA treated corneas in different groups (*n* = 6). (E) Representative OCT images showing corneal thickness changes mouse corneas (*n* = 6). (F) Corneal whole‐mount staining showing ATP1A1 expression in mouse CEnCs (*n* = 4). Scale bar: 30 µm. (G) Quantification of CCT (*n* = 6). The ^*^ represents the difference between UVA+DES and UVA+DES‐AAV‐*Foxo1* groups. H) Images of Alizarin Red‐stained of mouse CEnCs and quantification of cell density (*n* = 3). Scale bar: 50 µm. One‐way ANOVA (B,C,G,H) was used. ^*^ *p* < 0.05; ^***^ *p* < 0.001; ^****^ *p* < 0.0001; ns, not significant.

The protective effects and therapeutic potential of the DES‐AAV‐*Foxo1* intracameral system were further evaluated in both T1DM and FECD mouse models. In the T1DM model, DES‐AAV‐*Foxo1* significantly enhanced Na^+^/K^+^‐ATPase expression (Figure 8A) and decreased CCT (Figure 8B) compared with the DES‐only control. Notably, the number of CEnCs was significantly increased in the DES‐AAV*‐Foxo1*‐treated mice, reflecting the its capacity to promote cellular regeneration (Figure 8C). Similarly, in the UVA‐induced FECD model, slit‐lamp examination revealed that corneal edema was markedly alleviated in DES‐AAV‐*Foxo1*‐treated mice on days 1 and 3 following 500 J/cm^2^ UVA exposure (Figure 8D), accompanied by a significant reduction in CCT (Figure 8E,G). By day 7, corneal transparency was restored in all groups, and the CCT returning to the untreated baseline levels. At this timepoint, Na^+^/K^+^‐ATPase expression was elevated (Figure 8F), and CEnC density was markedly higher (Figure 8H), further supporting the protective role of this system. In summary, these in vivo findings demonstrate that the DES‐AAV‐*Foxo1* sustained‐release system robustly promotes structural and functional recovery of the corneal endothelium across in two distinct disease models, offering a promising non‐surgical strategy for the treatment and protection of corneal endothelial decompensation beyond conventional keratoplasty.

## 3 Conclusion

In the study, we engineered a DES‐based gene delivery system by incorporating AAV into the DES. The DES‐AAV‐*Foxo1* delivery system exhibited favorable biosafety, antioxidant bioactivity, restored mitochondrial function, and improved endothelial recovery. Notably, it enhanced AAV transduction efficiency compared to conventional methods and overcome current limitations of gene delivery in the anterior chamber. Furthermore, restoration of FOXO1 mitigates corneal endothelial dysfunction by improving mitochondrial‐ER contacts and maintaining mitochondrial Ca^2^^+^ homeostasis, manifested as preserved corneal transparency, decreased corneal thickness, and increased cell density. These findings highlighted the therapeutic potential of the DES‐AAV‐*Foxo1* delivery system in clinical treatment of corneal endothelial diseases.

Further researches were needed to broadly explore various functionalized DESs in treating a wide range of ocular disorders. First, the DES scaffold can be further functionalized with bioactive moieties, such as anti‐inflammatory cytokines, antioxidants, or matrix metalloproteinase (MMP) inhibitors, to provide synergistic cytoprotection alongside gene therapy. Second, optimizing the physicochemical properties of the DES—including particle size and surface charge—will allow for tailored administration via topical, subconjunctival, or intravitreal routes, enhancing bioavailability while minimizing off‐target effects. Crucially, clinical translation requires rigorous assessment of AAV immunogenicity and the long‐term performance of the DES carrier. Future studies should focus on the DES‐mediated protection of AAV from neutralizing antibodies and its degradation kinetics within the ocular microenvironment to ensure sustained therapeutic efficacy with minimal chronic inflammatory response. Collectively, these advancements will refine the DES‐AAV platform into a robust and biocompatible therapeutic strategy for diverse ocular disorders.

## 4 Experimental Section

### 4.1 Animals

Six‐ to eight‐week‐old male and female C57BL/6J mice were obtained from Vital River Laboratory Animal Technology (China). T1DM model was induced via a 5‐day course of intraperitoneal streptozotocin (STZ; Solarbio, Cat# IS250; 50 mg/kg) injections. Male mice with blood glucose levels greater than 16.7 mM were considered to have T1DM. FECD mouse model was established by female C57BL/6J mice. The corneas were exposed to UVA irradiation (500 J/cm^2^) for 20 min 57 s [66]. Animal experiments were conducted in accordance with the ARVO Statement for the Use of Animals in Ophthalmic and Vision Research and approved by the Ethics Committee of Eye Institute of Shandong First Medical University (No. SDSYKYJS 20231016).

### 4.2 Isolation of Nuclei From Mouse Corneas

Fresh corneas were minced in a 2 mL tube in lysis buffer with 1 mM DTT and 1 U/µL RNase inhibitor. The nuclei were extracted using the nucleus separation kit (CapitalBio Technology, Cat# XS0100101) and was subsequently filtered through a 40 µm cell strainer to remove larger cornea fragments. The nuclear pellet was resuspended in lysis buffer and resuspension buffer in a 2 mL tube after centrifugation and was subsequently subjected to density gradient centrifugation to separate the nuclei.

### 4.3 SnRNA‐seq and scATAC‐seq on the 10 × Genomics Platform

For snRNA‐seq, nuclei were isolated and used to prepare single‐nucleus libraries on the 10 × Genomics Chromium Single Cell 3’ platform, followed by sequencing on an Illumina NovaSeq 6000. For scATAC‐seq, nuclei were isolated and processed into libraries using the 10 × Genomics Chromium Single Cell ATAC solution, with subsequent sequencing performed on the Illumina NovaSeq 6000.

### 4.4 Data Processing and Downstream Analysis

For snRNA‐seq data, following alignment to the mouse reference genome (mm10) and count matrix generation with Cell Ranger (version 7.0.0), snRNA‐seq data were processed using Seurat (version 4.3.2) [71]. Nuclei with fewer than 500 genes or mitochondrial gene percentage greater than 20% were removed. DoubletFinder (version 2.0.4) was employed to detect and exclude doublets [72]. After integration [73, 74], normalization, feature selection and dimension reduction, the snRNA‐seq dataset were visualized via a UMAP plot. Differential gene expression was assessed using the “FindMarkers” function (|avg_log2FC| > 0.25, *p*‐value < 0.05). GO enrichment analysis of the DEGs was conducted by ClusterProfiler package and the results were displayed with the ggplot2 R package. For scATAC‐seq data, the FASTQ files were mapped to the mouse reference genome (mm10), and the peaks was called using Cell Ranger ATAC (version 2.1.0). Downstream analysis was processed with the Signac (version 1.13.0) pipeline [75]. Fewer than 1000 or greater than 30 000 fragments in peaks, fewer than 15% of reads mapping to peaks, fewer than 3 of TSS enrichment, and nucleosome signals greater than 4 were considered low‐quality nuclei. Ultimately, we acquired 16 855 high‐quality single nuclei for normalization, feature selection, and dimension reduction. UMAP was used for visualization, and differentially accessible regions were identified using the “FindMarkers” function with thresholds of |avg_log2FC| > 0.1 and *p*‐value < 0.05.

### 4.5 Bulk RNA‐Seq and Data Analysis

Total RNAs were isolated using KAPA Standard mRNA‐Seq Kit (Roche Diagnostics GmbH). cDNA libraries were constructed using the KAPA Stranded mRNA‐seq Library Kit for Illumina (Roche Diagnostics GmbH), followed by sequenced on the Illumina NovaSeq 6000 platform. After quality control and read mapping to the human reference genome (hg38), DEGs were identified using the limma package (version 3.58.1). The significance thresholds were defined as a *p*‐value < 0.05 and |avg_log2FC| > 0.585.

### 4.6 Gene Set Score Analysis

Gene set scores were analyzed by the “AddModuleScore” function in Seurat package. Gene sets were downloaded from the MSigDB database (https://www.gsea‐msigdb.org/gsea/msigdb). The results were displayed by the ggplot2 package.

### 4.7 Analysis of the Transcriptional Regulatory Network

The pyScenic workflow (version 1.10.0) was used to analyze the transcriptional regulatory networks of the DEGs [76]. The motif annotation databases used as references were obtained from https://resources.aertslab.org/cistarget/.

### 4.8 Transcription Factor (TF) Motifs Activity

TF motifs activity was estimated from scATAC‐seq data by the TFBSTools, and the “getMatrixSet” function was used to obtain DNA sequence information from JASPAR2020 motif dataset.

### 4.9 High‐Dimensional Weighted Gene Co‐Expression Network Analysis (hdWGCNA)

The gene co‐expression network between *Foxo1* and MAM‐related gene set using the R package WGCNA and hdWGCNA following the standard protocols provided in the respective packages [77].

### 4.10 Preparation of the DES‐Based AAV Delivery System

The DESs were prepared according to Zhu's reported method [78]. In detail, choline chloride and fructose with a certain ratio (2:1) were mixed in a round‐bottom flask, sealed, and heated at 80°C for 60 min under constant stirring. After cooling to room temperature, a homogeneous liquid was obtained. DES samples were filtered through a 0.22‐micron filter and stored at the room temperature. Before intracameral injection, AAV was mixed with DES to achieve a final concentration. The preparation should be used immediately after formulation. If stored at 4°C, it should be used within one week. Viral vectors (AAV2/6‐CMV‐*Foxo1*‐3Xflag‐P2A‐GdGreen‐tWPA) overexpressing *Foxo1* (NM_019739.3) and a negative control (AAV2/6‐CMV‐GdGreen‐tWPA) were purchased from OBiO Technology (China).

### 4.11 Intracameral AAV Delivery

The mice were anesthetized by pentobarbital sodium (40 mg/kg). Topical proparacaine hydrochloride eye drops were administered prior to the operation. An incision was made in the limbus using a pinhead of 11‐0 ophthalmic suture to create a tunnel into the anterior chamber, followed by the insertion of a 34‐gauge nanoneedle to inject 1 µL AAV or DES‐AAV solution.

### 4.12 Transmission Electron Microscopy (TEM)

Cornea samples were fixed with 2.5% glutaraldehyde overnight and 1% aqueous osmium tetroxide for 2 h. The samples were then dehydrated through a grade ethanol series, followed by infiltration and embedding in epoxy resin. Ultrathin sections (60 nm) were stained with 2% uranyl acetate and 2% lead citrate. Images were captured via a HT7700 Exalens transmission electron microscopy (HITACHI, Japan).

### 4.13 Corneal Endothelial Cell Density Analysis

The enucleated mouse corneas were stained with Alizarin Red S for 2 min and washed 3 times to remove excess stain. The corneal endothelium images were captured under a LEICA DM4 B light microscope (Leica, Germany). CEnC density was measured by Image J (WI, USA).

### 4.14 Cell Culture and Treatment

The human CEnC line (B4G12) was cultured in Human Endothelial SFM (Creative Bioarray, Cat# CM‐C3457L) supplemented with 3% FBS (OriCell, Cat# FBSSR‐01021‐50). For the high glucose‐induced corneal endothelial dysfunction, CEnCs were cultured in medium supplemented with 30 mM glucose, while 30 mM mannitol was used as osmotic control. For the H_2_O_2_‐induced corneal endothelial dysfunction, CEnCs were cultured in medium supplemented with 500 nM H_2_O_2_ for 12 h. For the UVA‐induced corneal endothelial dysfunction, CEnCs were exposed to 365 nm UVA (14.77 mW/cm^2^, Analytik Jena, XX‐15L) at a dose of 10 J/cm^2^ for 11 min 6 s at a distance of 10 cm [66].

### 4.15 Lentivirus Transduction

Lentiviral vectors for overexpressing *FOXO1* (NM_002015.4), and shRNA against *ITPR1* (sequence: 5’‐GCTCGGCATAACAAAGAACTT‐3’) and *FOXO1* (sequence: 5’‐CAGGACAATAAGTCGAGTTAT‐3’), were synthesized by GeneChem Biotechnology (China). CEnCs were transduced on day 2 at 30% confluence by adding fresh medium containing lentivirus overexpressing *FOXO1* (MOI = 10), *ITPR1* shRNA (MOI = 5) or *FOXO1* shRNA (MOI = 10).

### 4.16 Subcellular Fractionation

Cytosolic and nuclear proteins were extracted with a Nuclear and Cytoplasmic Extraction Kit (Beyotime, Cat# P0027). Isolation of subcellular fractions from cultured CEnCs was conducted following a previously described protocol [79]. The CEnCs were collected and homogenized in IBcells‐1 buffer. After centrifugation at 600 g for 5 min, the supernatant was centrifuged at 6000 g for 10 min. The supernatant was collected for ER isolation. The pellet was resuspended in IBcells‐2 buffer and centrifuged at 10 000 g for 10 min. Then, the pellet was collected and resuspended in mitochondrial resuspension buffer (MRB). Mitochondria and the MAM were separated from the crude mitochondria with percoll medium via ultracentrifugation at 95 000 g for 30 min. Finally, the upper layer containing MAM and the lower layer containing mitochondria were resuspended in MRB. The pure mitochondria were collected after centrifugation at 6300 g for 10 min. The MAM fraction was extracted via ultracentrifugation at 100 000 g for 1 h.

### 4.17 Western Blotting

The isolated subcellular fractions, cultured CEnCs, and mouse CEnCs samples were lysed in RIPA buffer. The extracted protein was separated via SDS‐PAGE, and immunoreactive bands were detected with a ECL detection kit. The primary antibodies used are detailed in Table S2.

### 4.18 Chromatin Immunoprecipitation (ChIP)

ChIP assays were conducted using a Pierce Magnetic ChIP Kit (Thermo, Cat# 26157). After crosslinking the cells with formaldehyde, we extracted the nuclei of CEnCs and using micrococcal nuclease to fragment the chromatin. The fragmented DNA/protein complex was incubated with antibodies specific for FOXO1. Finally, the DNA was purified and analyzed via qPCR. The primers and antibodies were listed in Tables S1 and S2.

### 4.19 Co‐Immunoprecipitation (Co‐IP)

The collected CEnCs were lysed and incubated with a primary antibody specific for GRP75 on the shaker overnight at 4°C. Then, the lysates were mixed with 15 µL of magnetic beads (Beyotime, Cat# P2018) and incubated on the shaker for 6 h at 4°C. The mixture was washed with TBS, and the protein was eluted with 1 × loading buffer for subsequent immunoblotting.

### 4.20 Fluorescent Staining of Mitochondria and the ER

CEnCs were cultured in HBSS supplemented with 200 nM Mito‐Tracker Green (Beyotime, Cat# C1048), 1 µM ER‐Tracker Red (Invitrogen, Cat# E34250) and Hoechst 33342 (Solarbio, Cat# C0031) for 30 min at CO_2_ incubator. The cells were viewed under a Zeiss LSM880 confocal microscope (Carl Zeiss, Germany).

### 4.21 In Situ Proximity Ligation Assay (PLA)]

The IP3R1 and VDAC1 interactions were detected using the Duolink in Situ Detection Kit (Sigma–Aldrich, Cat# DUO92002/4/8). After fixation, permeabilization and blocking, the CEnCs and mice corneas were incubated with primary antibodies. After removing primary antibodies, the CEnCs and mice corneas were probed with the secondary antibodies linked to oligonucleotides, followed by ligation and signal amplification. The fluorescence signals were captured by a Zeiss LSM880 confocal microscope (Carl Zeiss, Germany).

### 4.22 Mitochondrial Ca^2+^ and MitoSOX Staining

CEnCs were cultured in serum free medium containing 5 µM Rhod‐2 AM (Abcam, Cat# ab142780) and 200 nM Mito‐Tracker Green (Beyotime, Cat# C1048) for 35 min at 37°C. CEnCs were cultured in HBSS containing 2 µM MitoSOX (Yeason, Cat# 40778ES50) for 20 min at 37°C. Fluorescence images were captured with a LEICA DMi8 inverted microscope (Leica, Germany).

### 4.23 Mitochondrial Oxygen Consumption Rate (OCR) Measurements

The OCR was measured by a Seahorse XFp Cell Mito Stress Test Kit (Agilent Technologies AG, Switzerland). In brief, the CEnCs were cultured in 8‐well Seahorse XFp microplates and incubated for 24 h. Oxygen consumption was measured after incubated sequentially with 1.5 mM oligomycin, 2 mM FCCP and 0.5 mM rotenone/antimycin A.

### 4.24 Evaluation of Mitochondrial Respiratory Chain (MRC) Activity

Isolated mitochondria of human CEnCs were used to measure mitochondrial electron transport chain complex activities. Complex I‐V activities were determined by microplate assay kits (Solarbio, Cat# BC0515; Cat# BC3235, Cat# BC3245, Cat# BC0945, Cat# BC1445) and measurements were taken with a Multiskan FC spectrophotometer (Thermo, USA).

### 4.25 Statistical Analysis

Single‐nuclei transcriptomic data was analyzed by R (version 4.3.2). Statistical analysis was conducted with Wilcoxon rank‐sum test. *p* < 0.05 were regarded as statistically significant. Experimental data was analyzed by the Graphpad Prism (Version 10.0.2). Statistical analysis was conducted with Student's *t*‐test or one‐way ANOVA. The values are presented as the mean ± standard deviation (SD). *p* < 0.05 were regarded as statistically significant.

## Author Contributions

H.Z. performed investigation and formal analysis; H.Z. S.D., H.W., and Z.L. wrote the manuscript; H.W., Z.L., Q.W., X.J., Q.Z., S.D., and L.X. performed conceptualization; H.Z., X.L., and Z.L. performed methodology; H.Z. and X.L. performed software, data curation and validation; H.Z., X.L., and S.D. performed visualization; H.W., Y.Z., X.Q., Q.Z., and L.X. performed resources; S.D. and Q.Z. reviewed the manuscript; Q.W., S.D., and L.X. supervised the project; L.X. and S.D. acquired the funding support. All authors read and approved the final manuscript.

## Conflicts of Interest

The authors declare no conflicts of interest.

## Supporting information




**Supporting File 1**: advs75464‐sup‐0001‐SuppMat.docx.

**Supporting File 2**: advs75464‐sup‐0002‐Data.zip.

## Data Availability

Mouse snRNA‐seq and scATAC‐seq data have been deposited in the Genome Sequence Archive (GSA) of China National Center for Bioinformation (CNCB) under the accession number CRA021806 and CRA021694 [80]. Bulk RNA‐seq data have been deposited in Gene Expression Omnibus (GEO) repository under the accession number GSE325372.
