# Supplementary material for: A Viscous DES‐AAV‐Foxo1 Delivery System With High Transfection Efficiency for the Treatment of Corneal Endothelial Dysfunction by Restoring Mitochondria‐ER Contacts
Source: Adv Sci (Weinh). 2026 May 3;13(41):e75464. doi: 10.1002/advs.75464 (PMC13335468; doi:10.1002/advs.75464)
Supplement: Supplementary file 1 — Supporting File 1: advs75464‐sup‐0001‐SuppMat.docx. [file ADVS-13-e75464-s001.docx]

Supporting Information

**A viscous DES-AAV-*Foxo1* delivery system with high transfection efficiency for the treatment of corneal endothelial** **dysfunction by restoring mitochondria-ER contacts**

*Hongran Zhao^1,2,3^, Xiaoyu Li^1,2^, Hongwei Wang^1,2^, Zongyi Li^1,2^, Qun Wang^1,2^, Yangyang Zhang^1,2^, Xu Jing^4^, Xia Qi^1,2^, Qingjun Zhou^1,2,*^, Shengqian Dou^1,2,*^ and Lixin Xie^1,2,*^*

**This file includes**:

1. Supplementary Figures 1 to 11

2. Supplementary Tables 1 to 2

3. Description of Supplementary Datasets 1 to 6

1. Supplementary Figures


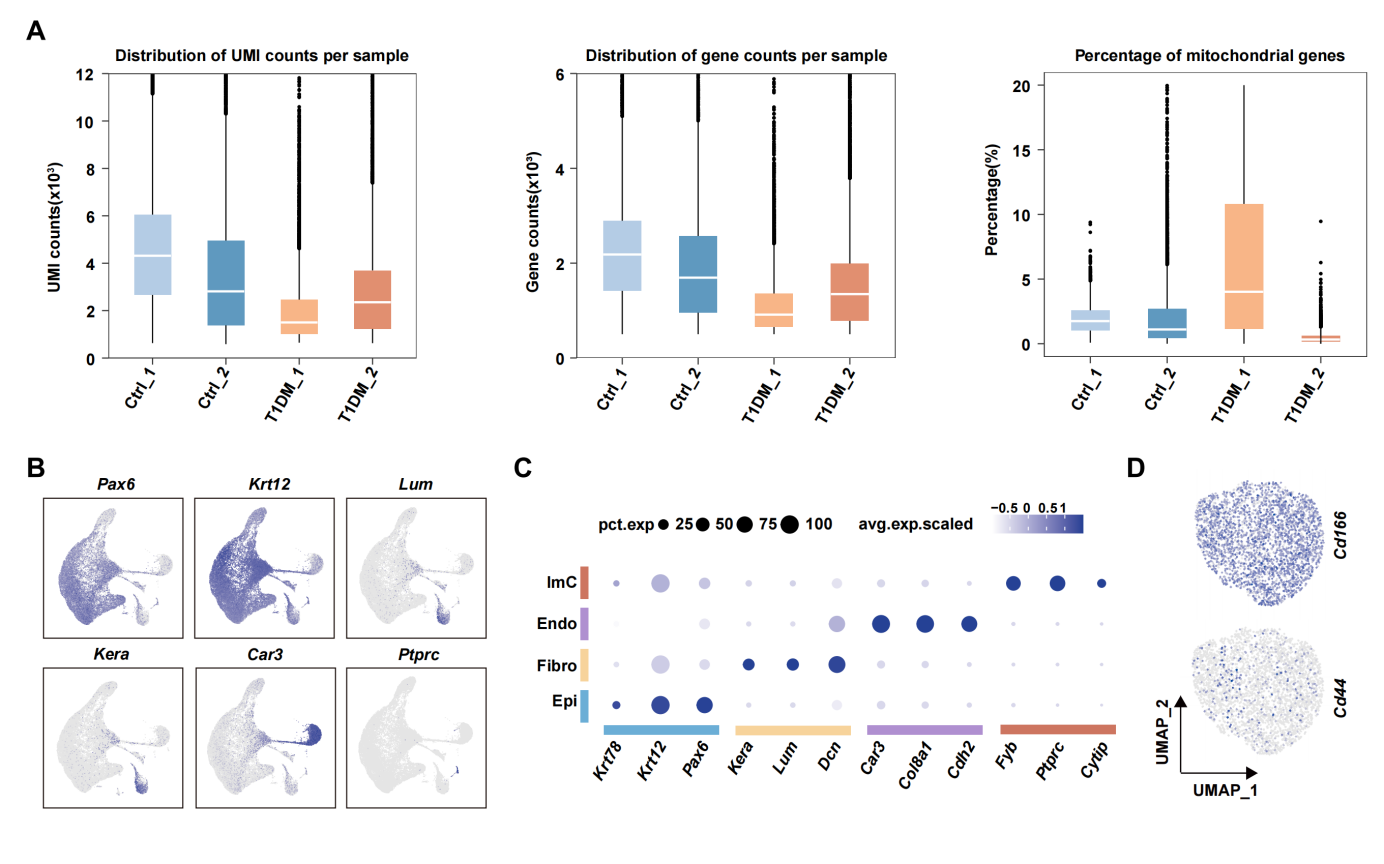


**Figure S1.** Quality control of mouse snRNA-seq. A) Box plots showing the number of UMI counts, gene numbers, and the percentages of mitochondrial genes per cell for different snRNA-seq samples. B) Feature plots depicting the representative marker genes for each cell type. C) Dot plot depicting the expression of established marker genes in mouse cornea. D) Feature plots showing the *CD166*^+^ cells (upper) and *CD44*^-^ cells (lower) among CEnCs.


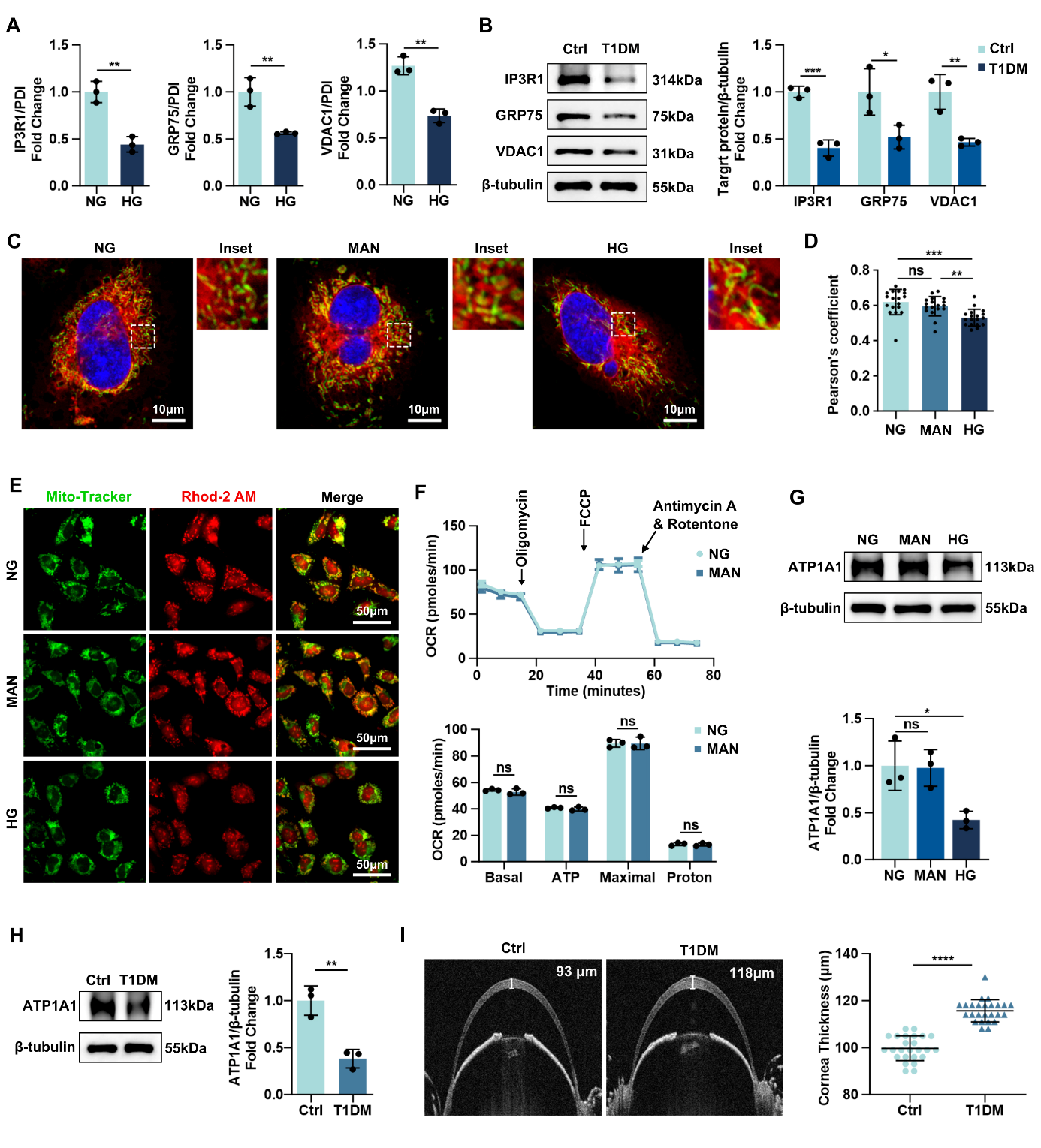
Figure S2. Impairment of MAM and pump function in diabetic corneal endothelium. A) Quantitative analysis of IP3R1, GRP75 and VDAC1 protein levels in MAM fractions (n = 3). NG: normal glucose; HG: high glucose. B) WB analysis of IP3R1, GRP75 and VDAC1 expression levels in CEnCs of control and T1DM mice (n = 3). C) Confocal images displaying the associations between the ER (ER-Tracker Red) and mitochondria (Mito-Tracker Green) in human CEnCs cultured under NG, MAN (Mannitol) and HG conditions (n = 3). Scale bar: 10 μm. D) Correlation analysis of ER and mitochondrial contacts using Pearson’s coefficient (n = 3). E) Mitochondrial Ca^2+^ levels were determined by Rhod-2 AM staining in human CEnCs (n = 3). Scale bar: 50 μm. F) Mitochondrial oxygen consumption rate (OCR) measurements were performed to assess basal respiration, ATP-dependent respiration, maximal respiration and proton leakage in NG- and MAN-treated CEnCs (n = 3). G) WB analysis of ATP1A1 expression levels in the CEnCs cultured under NG, MAN or HG conditions (n = 3). H) WB analysis of ATP1A1 expression levels in the CEnCs from control and T1DM mice (n = 3). I) Representative optical coherence tomography (OCT) images of corneas from control and T1DM mice and quantification analysis of central corneal thickness (CCT) (n = 20). Student’s *t*-test (A, B, F, H, I) and one-way ANOVA (D, G) were used. ***p* < 0.01; ****p* < 0.001; *****p* < 0.0001; ns, not significant.

**
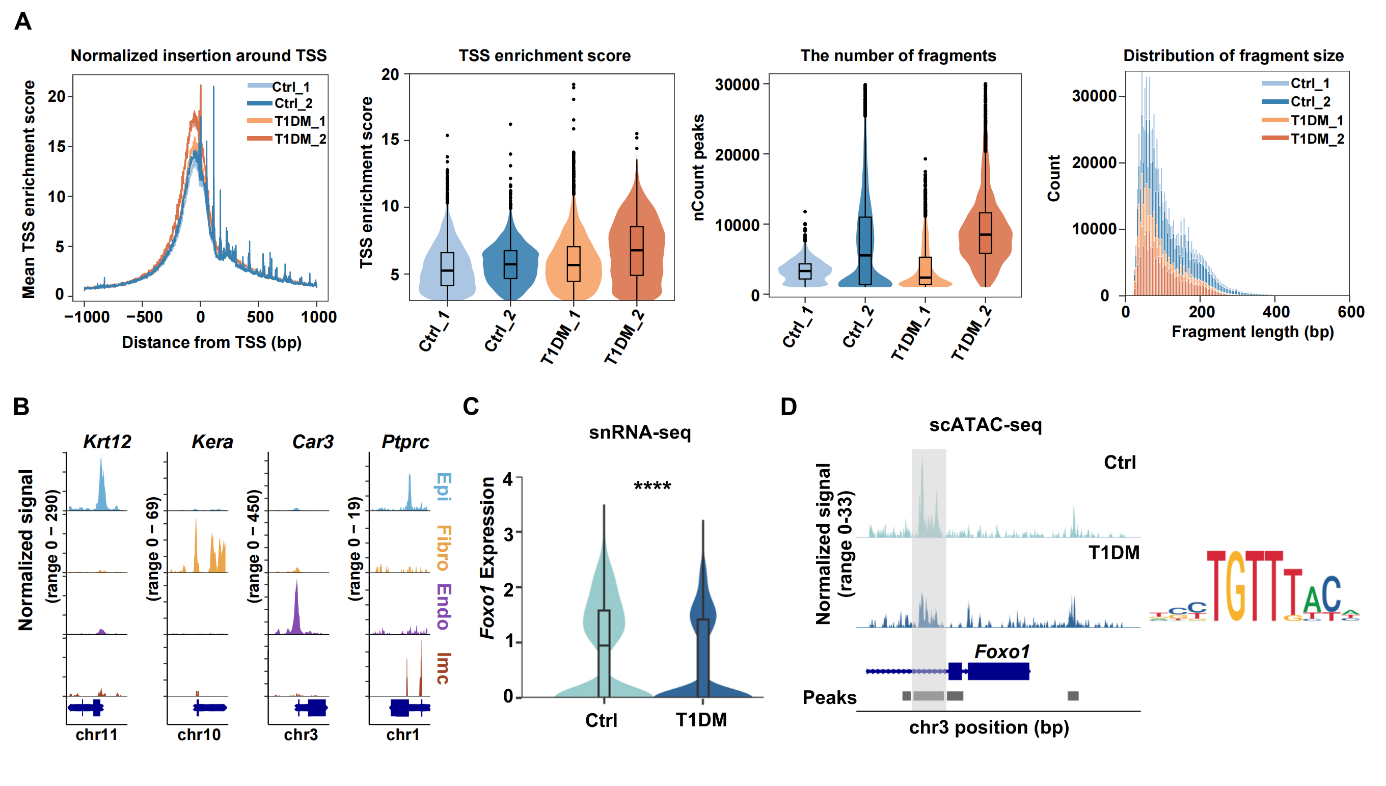
**

**Figure S3.** Integrated analysis of scATAC-seq and snRNA-seq data. A) Plots depicting the normalized insertion profile distribution around the transcription start site (TSS), TSS enrichment, the number of fragments, and fragment distribution of different scATAC-seq samples. B) Coverage plots depicting the chromatin accessibility of known cell type marker genes of the mouse scATAC-seq data. C) Violin plots depicting the *Foxo1* expression in the nuclei of CEnCs between Ctrl and T1DM groups by snRNA-seq. D) Coverage plot depicting the chromatin accessibility tracks of *Foxo1* in the nuclei of CEnCs between control and T1DM groups by scATAC-seq. The *Foxo1* binding motif is shown as a motif logo on the right. Wilcoxon rank-sum test (C) was used. **** *p* < 0.0001.


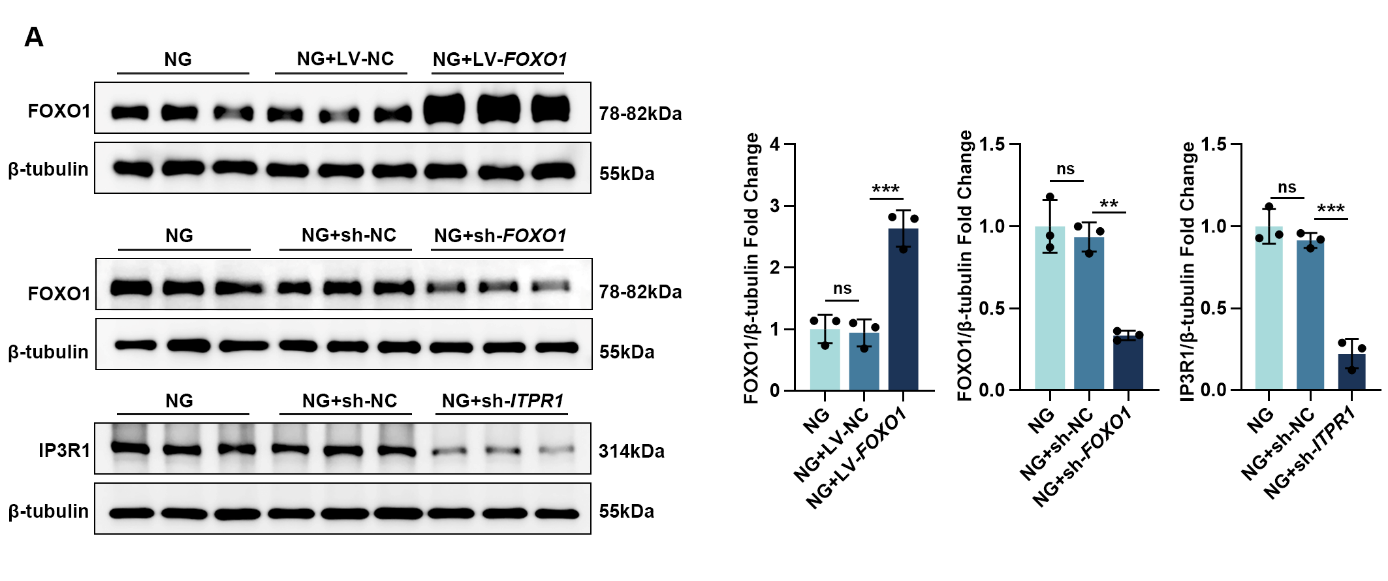


**Figure S4.** The transfection efficiency of lentivirus. A) WB analysis the expression of FOXO1 and IP3R1 in human CEnCs (n=3). One-way ANOVA was used. ***p* < 0.01; ****p* < 0.001; ns, not significant.

**
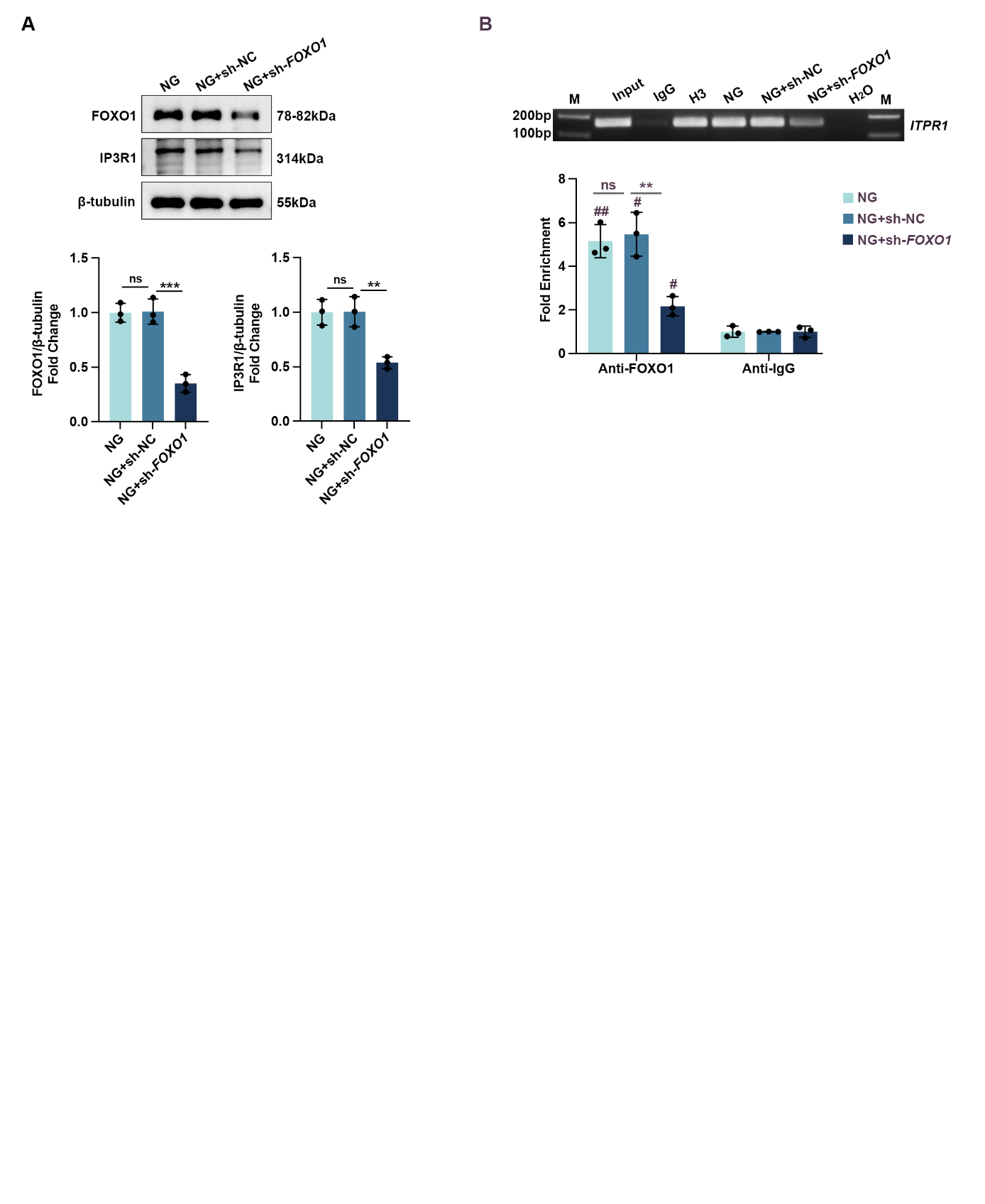
 Figure S5.** Validation of FOXO1-dependent regulation of IP3R1. A) WB analysis the expression of FOXO1 and IP3R1 in human CEnCs (n=3). B) ChIP assays verified the binding of FOXO1 to the *ITPR1* promoter region in human CEnCs between NG+sh-NC and NG+sh-*FOXO1* groups (n = 3). One-way ANOVA (*, between groups) and Student’s *t*-test (^#^, compared with the corresponding IgG) were used. ***p* < 0.01; ****p* < 0.001; ^#^*p* < 0.05; **^##^***p* < 0.01; ns, not significant.


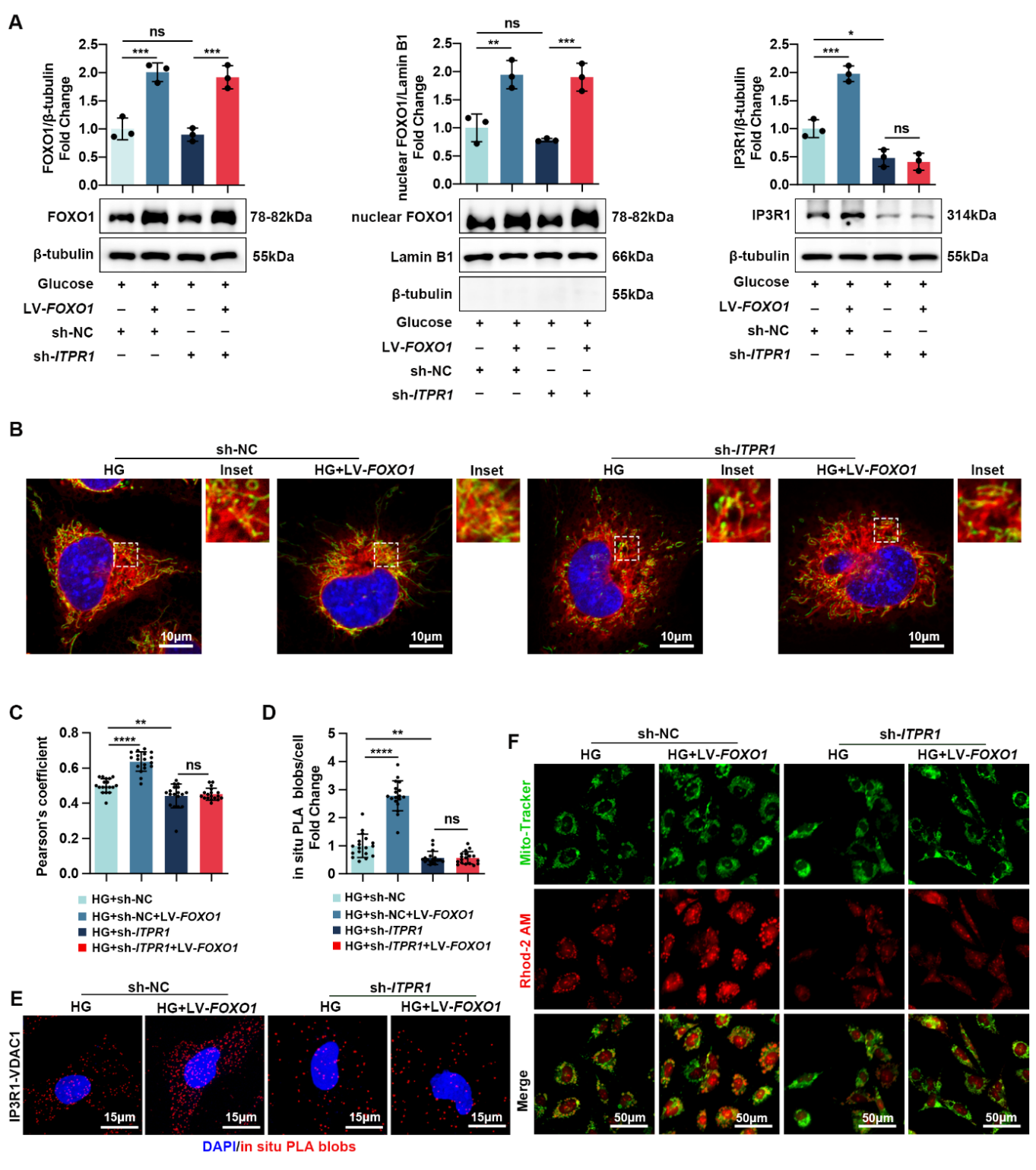


**Figure S6.** FOXO1 promotes MAM formation and Ca^2+^ transport by up-regulating IP3R1. A) WB analysis of FOXO1 and IP3R1 protein levels in human CEnCs treated with HG + sh-NC, HG + sh-NC + LV-*FOXO1*, HG + sh-*ITPR1*, or HG + sh-*ITPR1* + LV-*FOXO1* (n = 3). LV: Lentivirus. B) Confocal images displaying the associations between the ER (ER-Tracker Red) and mitochondria (Mito-Tracker Green) in cultured human CEnCs. Scale bar: 10 μm. C) Correlation analysis of ER and mitochondrial contacts using Pearson’s coefficient (n = 3). D-E) Representative images of the PLA showing the interaction between the MAM tethering proteins IP3R1 and VDAC1 (n = 3). Scale bar: 15 μm. F) Mitochondrial Ca^2+^ levels were determined via Rhod-2AM staining of human CEnCs (n = 3). Scale bar: 50 μm. One-way ANOVA (A, C, D) was used. **p* < 0.05; ***p* < 0.01; ****p* < 0.001; *****p* < 0.0001; ns, not significant.

**
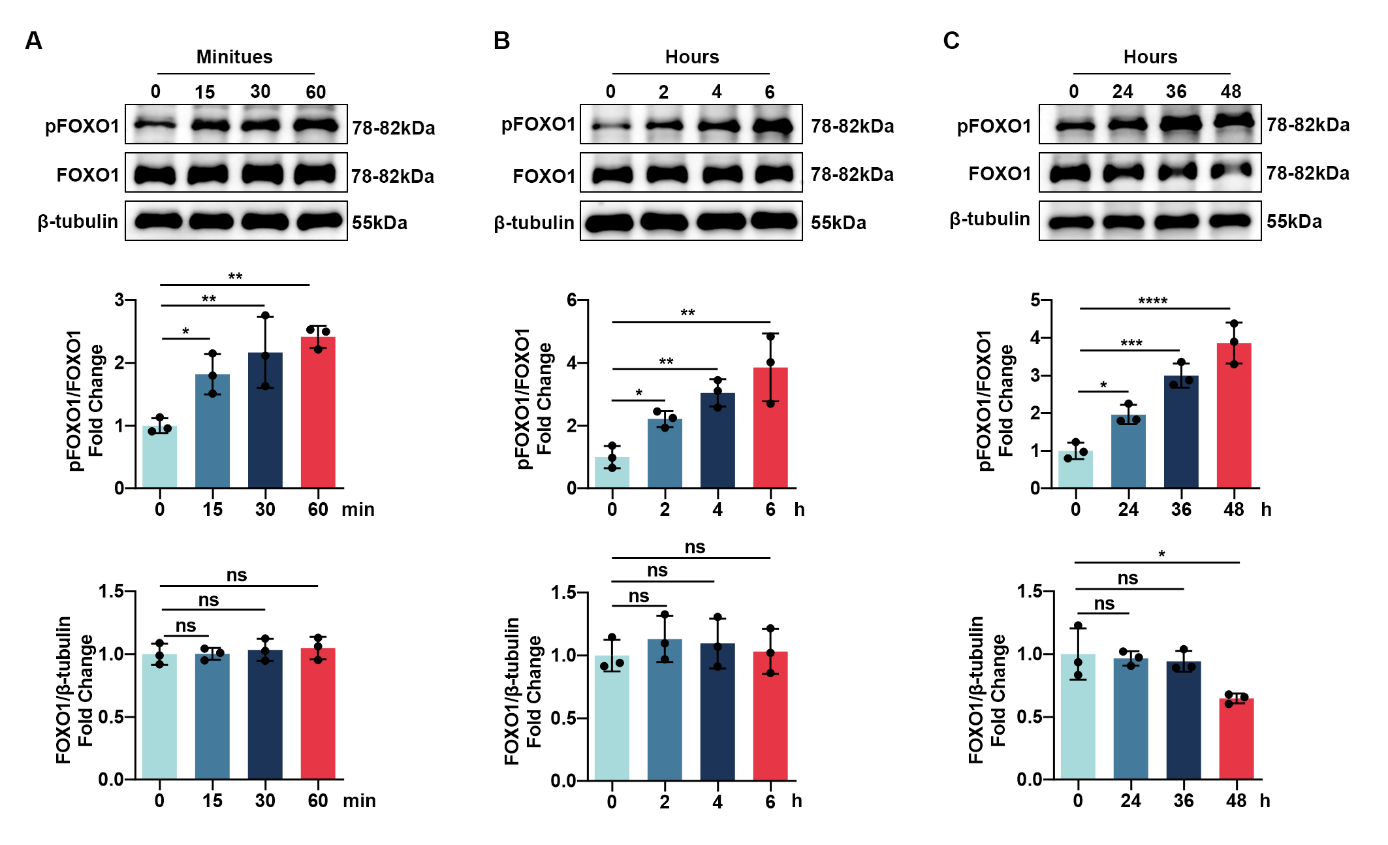
**

**Figure S7.** The phosphorylation of FOXO1 at Ser256. A-C) WB analysis of FOXO1 phosphorylated at Ser256 and total FOXO1 protein levels at different time points (n = 3). One-way ANOVA (A, B, C) was used. **p* < 0.05; ***p* < 0.01; ****p* < 0.001; *****p* < 0.0001; ns, not significant.


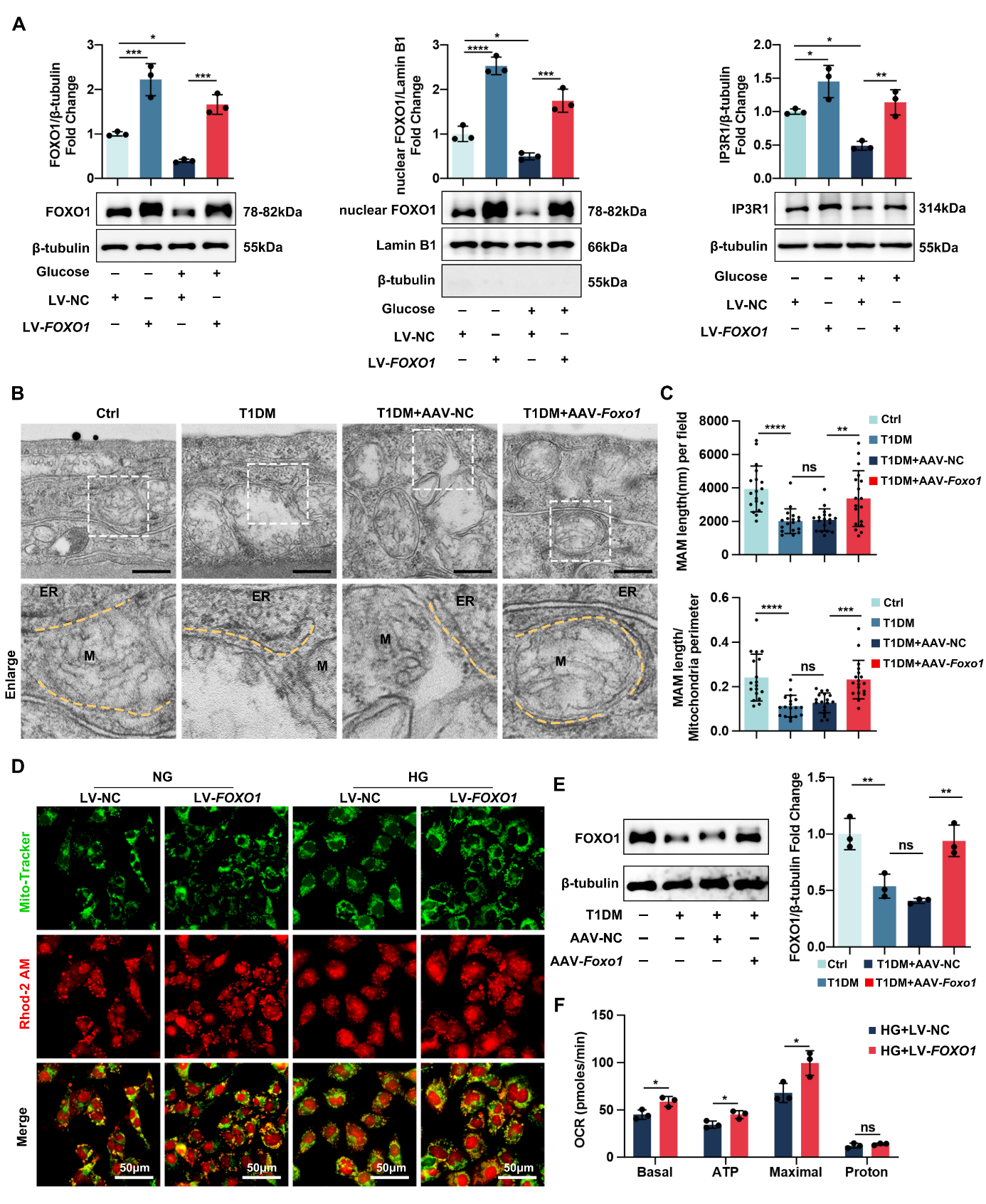


**Figure S8.** FOXO1 increases mitochondria-ER contacts and promotes Ca^2+^ transport. A) WB analysis of FOXO1 and IP3R1 protein levels in human CEnCs (n = 3). LV: Lentivirus. B) TEM images showing the ultrastructure of the corneal endothelium in control mice, mice with T1DM, mice with T1DM treated with AAV-NC, and mice with T1DM treated with AAV-*Foxo1* (n = 3). Scale bar: 500 nm. The yellow dotted line represents the MAM interface. AAV: Adeno-associated virus. C) Quantification of total MAM length per field and the length of the ER adjacent to the mitochondria, normalized to total mitochondria length (n = 3). D) Mitochondrial Ca^2+^ levels were determined by Rhod-2 AM staining in human CEnCs (n = 3). Scale bar: 50 μm. E) WB analysis of FOXO1 protein levels in mouse CEnCs. F) OCR measurements were performed to assess basal respiration, ATP-dependent respiration, maximal respiration, and proton leakage in the HG + LV-NC and HG + LV-*FOXO1* groups (n = 3). Student’s *t*-test (F) and one-way ANOVA (A, C, E) were used. **p* < 0.05; ***p* < 0.01; ****p* < 0.001; *****p* < 0.0001; ns, not significant.


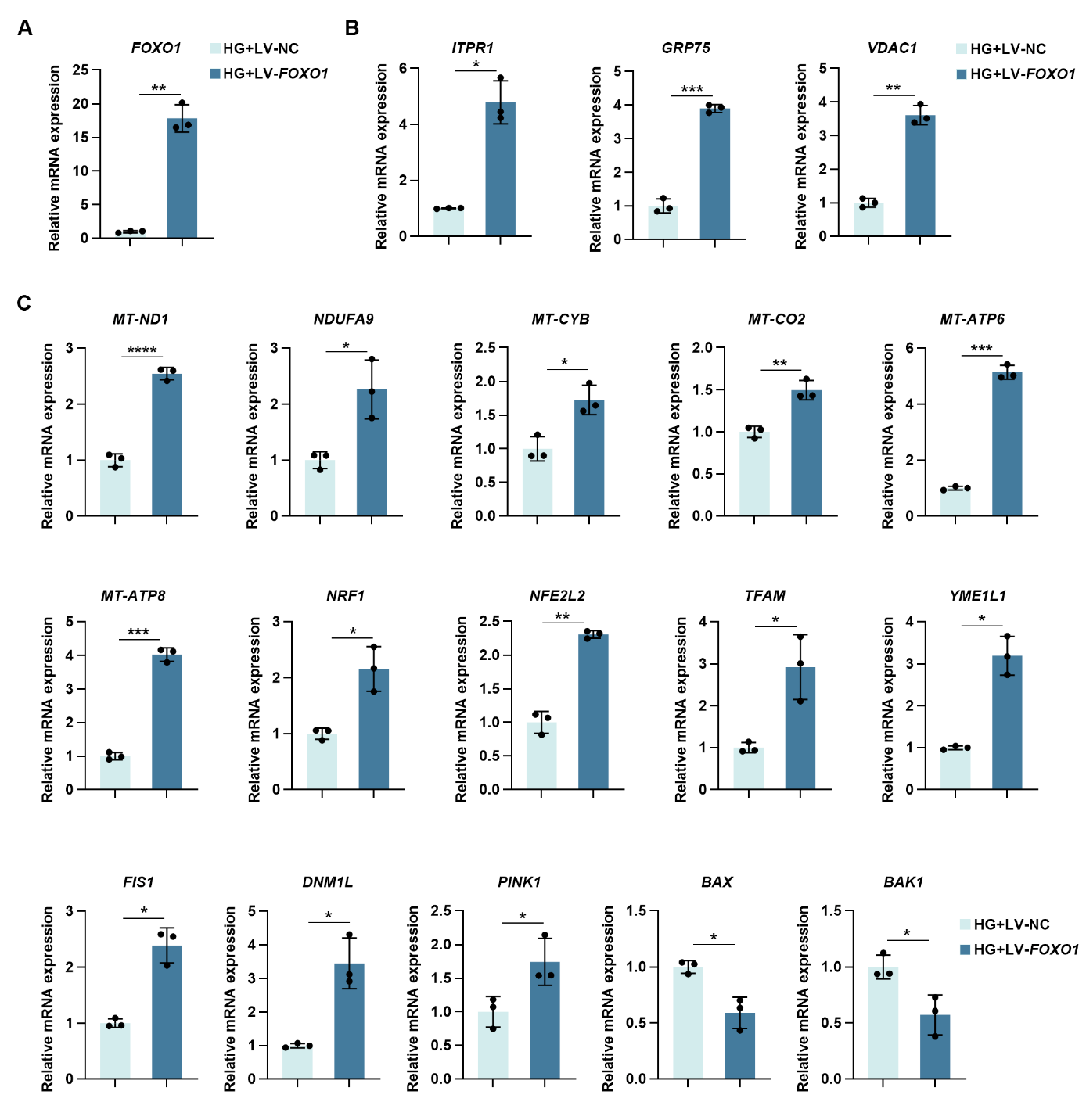


**Figure S9.** FOXO1 overexpression restores HG-induced mitochondrial damage. A) qPCR analysis of *FOXO1* mRNA expression in HCEnCs. B) qPCR analysis of mRNA expression of *ITPR1*, *GRP75*, *VDAC1* in HCEnCs. C) qPCR analysis of mRNA expression of genes associated with mitochondrial function and homeostasis in HCEnCs. Student’s *t*-test was used. **p* <0.05; ***p* < 0.01; ****p* < 0.001; *****p* < 0.0001.


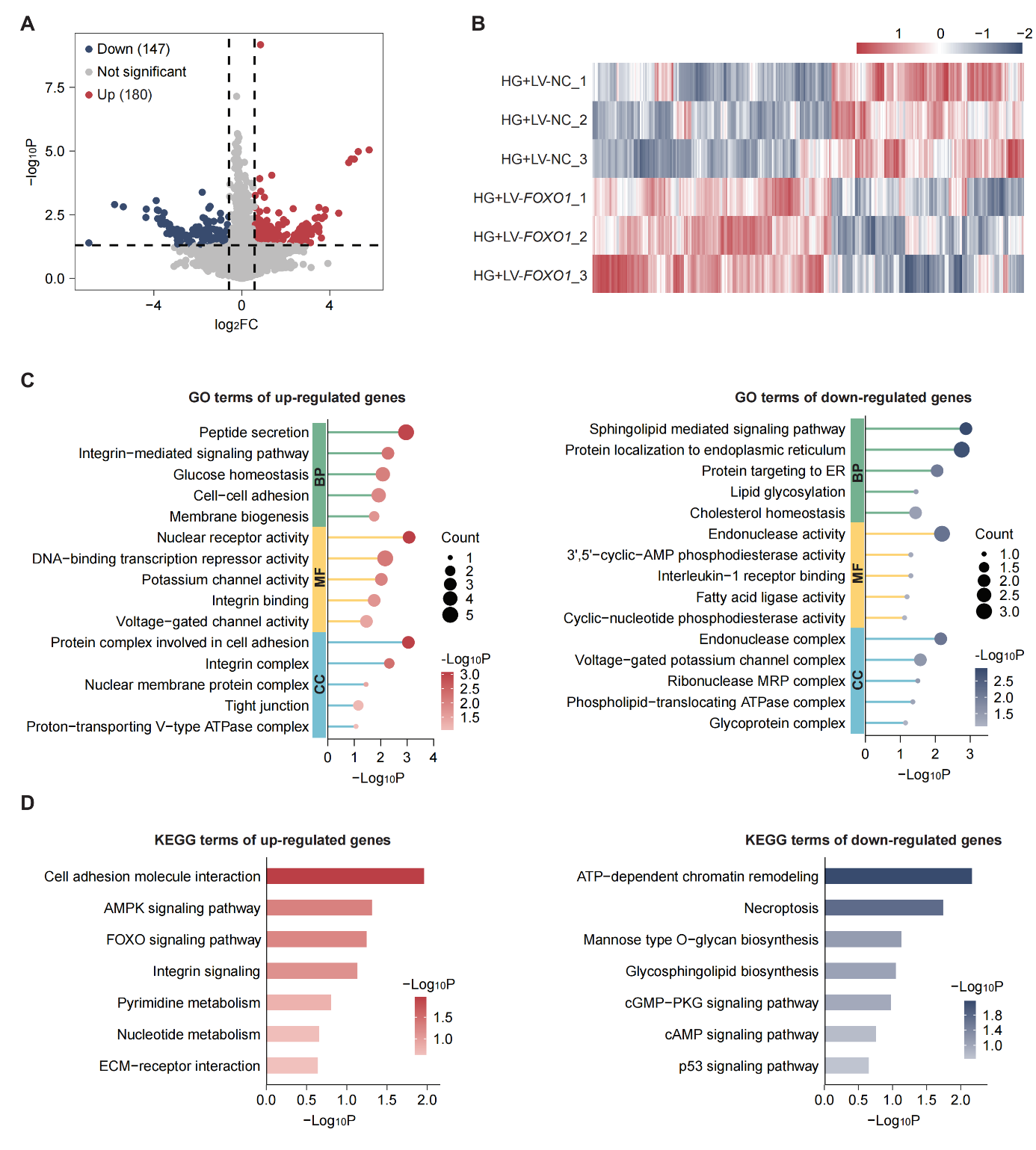


**Figure S10.** Transcriptomic impact of *FOXO1* overexpression. A) Volcano plot showing the distribution of differentially expressed genes (DEGs) in *FOXO1*-overexpressing HCEnCs compared to negative control under HG treatment. B) Heatmap showing the DEG expression across samples. C) Representative GO terms of the up‐ and down-regulated genes. D) Representative KEGG terms of the up- and down- regulated genes.


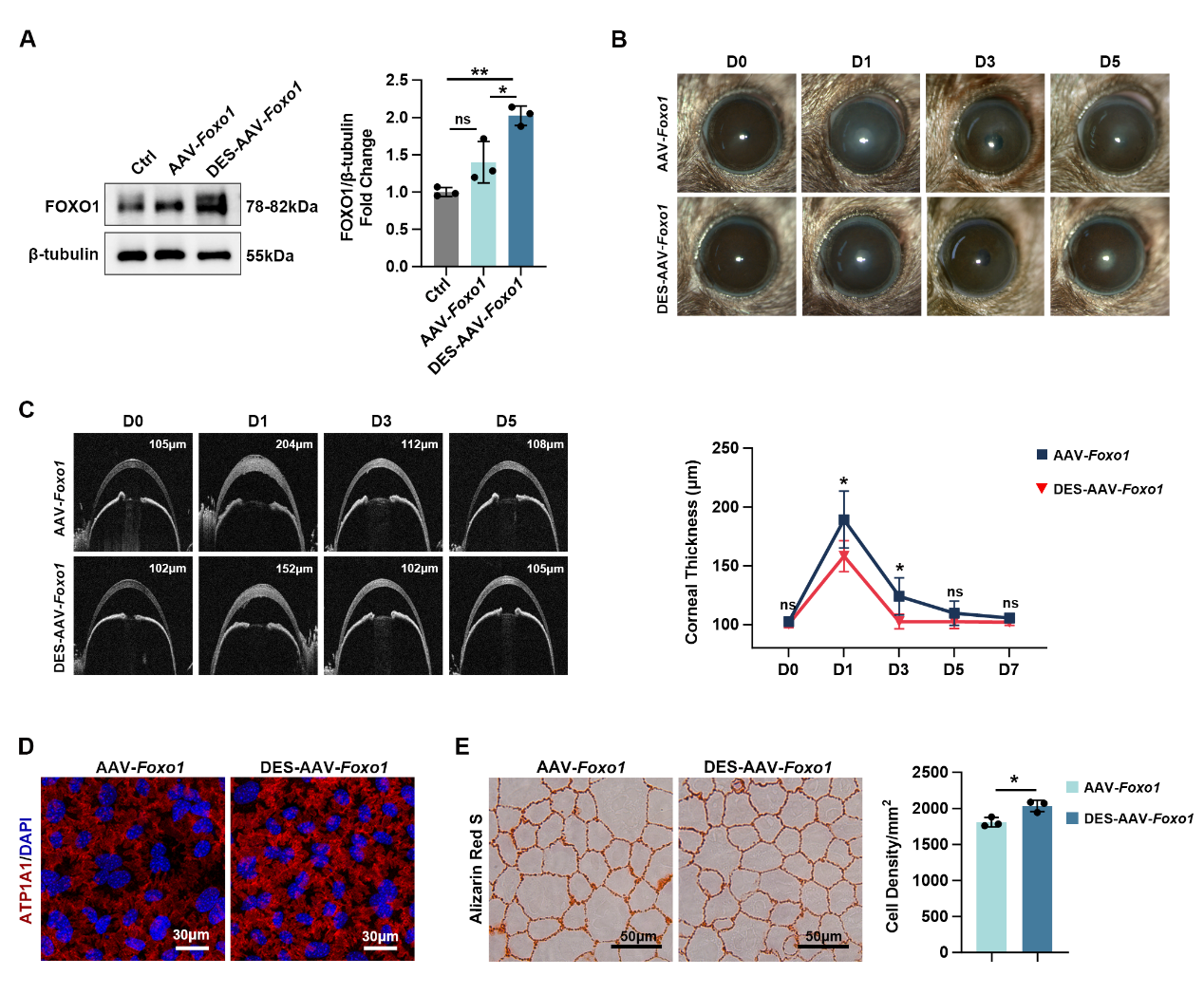


**Figure S11.** DES-AAV-*Foxo1* delivery system enhances AAV transfection efficiency and rescues corneal endothelial dysfunction. A) WB analysis of FOXO1 expression in mouse CEnCs (n = 3). B) Representative slit-lamp images showing the changes in pre- or post-UVA treated corneas in different groups (n = 6). C) Representative OCT images of the mouse cornea and quantification of CCT (n = 6). D) Corneal whole-mount immunofluorescence showing the ATP1A1 expression in mouse CEnCs (n = 4). Scale bar: 30 μm. E) Images of Alizarin Red-stained of mouse CEnCs and quantification of endothelial cell density (n = 3). Scale bar: 50 μm. One-way ANOVA (A) and Student’s *t*-test (C, E) were used. **p* <0.05; ***p* < 0.01; ns, not significant.

1. **Supplementary Tables**

**Table S1. Primers used in this study.**

| Primers | Sequence (5’-3’) |
| --- | --- |
| *FOXO1*-Forward | ACGCTGTCGCAGATCTACGA |
| *FOXO1*-Reverse | AGTTCCTTCATTCTGCACACGAA |
| *ITPR1*-Forward | TGTTCGGCTCAGACACCTATGTA |
| *ITPR1*-Reverse | GCTGGCATCATTGGCAAAGT |
| *GRP75*-Forward | AAGATGGAAGAATTCAAGGACCAA |
| *GRP75*-Reverse | TGATGCCTGCTGAAGAGAGGAT |
| *VDAC1*-Forward | GCTCAGGCTCAGCCAACACT |
| *VDAC1*-Reverse | GTGCAAGCTGATCTTCCACAGTAA |
| *MT-ND1*-Forward | CAAAGGCCCCAACGTTGTAG |
| *MT-ND1*-Reverse | AGAAGAGCGATGGTGAGAGCTAA |
| *NDUFA9*-Forward | CCAAGGAAGCTGGAGTTGAAA |
| *NDUFA9*-Reverse | TGTCCGACGGCTTTACGATAA |
| *MT-CYB*-Forward | GCAATAATCCCCATCCTCCATAT |
| *MT-CYB*-Reverse | TCCAATGATGGTAAAAGGGTAGCT |
| *MT-CO2*-Forward | CTCATGAGCTGTCCCCACATT |
| *MT-CO2*-Reverse | AGGACGATGGGCATGAAACT |
| *MT-ATP6*-Forward | CGCCGCAGTACTGATCATTCT |
| *MT-ATP6*-Reverse | GTTCGTCCTTTAGTGTTGTGTATGGT |
| *MT-ATP8*-Forward | ATACTACCGTATGGCCCACCAT |
| *MT-ATP8*-Reverse | CATTTTGGTTCTCAGGGTTTGTTATAA |
| *NRF1*-Forward | TCAAGTACTCTACAGGTCGGGGA |
| *NRF1*-Reverse | TTCCCGCCCATGCTGTTTA |
| *NFE2L2*-Forward | AGCATGCCCTCACCTGCTA |
| *NFE2L2*-Reverse | TGAAATGCCGGAGTCAGAATC |
| *TFAM*-Forward | TTTACCGAGGTGGTTTTCATCTG |
| *TFAM*-Reverse | CGCTGGGCAATTCTTCTAATTAG |
| *YME1L1*-Forward | AAACTGGTTTTGCGGAAGGTT |
| *YME1L1*-Reverse | GGAAGCGGACAGATAAAAATGG |
| *FIS1*-Forward | CTGGTGCGGAGCAAGTACAA |
| *FIS1*-Reverse | TAAGGCCTTCTCGTATTCCTTGA |
| *DNM1L*-Forward | GCGGCAAATCAAACGTCTAGAA |
| *DNM1L*-Reverse | TTTGTAACAGGCAACCTTTTACGA |
| *PINK1*-Forward | TCCCCTTGGCCATCAAGAT |
| *PINK1*-Reverse | GGGACCTCTCTTGGATTTTCTGT |
| *BAX*-Forward | TGGCAGCTGACATGTTTTCTG |
| *BAX*-Reverse | TCCCGGAGGAAGTCCAATG |
| *BAK1*-Forward | CGCTTCGTGGTCGACTTCAT |
| *BAK1*-Reverse | TACCACAAACTGGCCCAACA |
| *ACTB*-Forward | GGGAAATCGTGCGTGACATT |
| *ACTB*-Reverse | GGAACCGCTCATTGCCAAT |
| *ITPR1*-Forward (ChIP) | GGGAGAGAGGCTATTTAAGGCAAT |
| *ITPR1*-Reverse (ChIP) | GCACAATCCAGGGCTCAGA |

**Table S2. Antibodies used in this study.**

| Antibodies | Source | Identifier |
| --- | --- | --- |
| Mouse monoclonal anti-IP3R1 | Santa Crus | Cat# sc-271197 |
| Rabbit monoclonal anti-GRP75 | Cell Signaling Technology | Cat# 3593 |
| Rabbit monoclonal anti-VDAC1 | Proteintech | Cat# 55259-1-AP |
| Mouse monoclonal anti-COXIV | Proteintech | Cat# 66110-1-Ig |
| Mouse monoclonal anti-PDI | Proteintech | Cat# 66422-1-Ig |
| Mouse monoclonal anti-β-tubulin | Proteintech | Cat# 66240-1-Ig |
| Mouse monoclonal anti-Lamin B1 | Proteintech | Cat# 66095-1-Ig |
| Rabbit monoclonal anti-pFOXO1 | Cell Signaling Technology | Cat# 84192 |
| Rabbit monoclonal anti-FOXO1 | Cell Signaling Technology | Cat# 2880 |
| Rabbit anti-IgG | Beyotime | Cat# A7016 |
| Mouse monoclonal anti-ZO-1 | Thermo Fisher Scientific | Cat# 33-9100 |
| Rabbit monoclonal anti-ATP1A1 | Abcam | Cat# ab76020 |
| Alexa Flour 488 Goat Anti-Rabbit IgG H+L | Abcam | Cat# ab150077 |
| Alexa Flour 647 Donkey Anti-Rabbit IgG H+L | Abcam | Cat# ab150075 |
| Alexa Flour 488 Goat Anti-Mouse IgG H+L | Abcam | Cat# ab150113 |

1. **Description of Supplementary Datasets 1 to 6**

**Dataset S1.** Marker genes for each cell type of mouse cornea in snRNA-seq data.

**Dataset S2.** Differentially expressed genes (DEGs) identified in corneal endothelium between Ctrl and T1DM mice.

**Dataset S3.** The gene sets required for gene set score analysis.

**Dataset S4.** Differentially expressed genes (DEGs) of each cell type of mouse cornea in scATAC-seq data.

**Dataset S5.** Differentially accessible peaks (DAPs) identified in corneal endothelium between Ctrl and T1DM mice.

**Dataset S6.** Differentially expressed genes (DEGs) identified in HCEnCs between HG+LV-NC and HG+LV-*FOXO1* groups.
